# Neurotransmitter Switching Coupled to β-Adrenergic Signaling in Sympathetic Neurons in Prehypertensive States

**DOI:** 10.1161/HYPERTENSIONAHA.118.10844

**Published:** 2018-05-09

**Authors:** Emma N. Bardsley, Harvey Davis, Keith J. Buckler, David J. Paterson

**Affiliations:** From the Wellcome Trust OXION Initiative in Ion Channels and Disease, Burdon Sanderson Cardiac Science Centre, Department of Physiology, Anatomy and Genetics, University of Oxford, United Kingdom.

**Keywords:** cardiovascular diseases, epinephrine, hypertension, sequence analysis, RNA, stellate ganglion

## Abstract

Single or combinatorial administration of β-blockers is a mainstay treatment strategy for conditions caused by sympathetic overactivity. Conventional wisdom suggests that the main beneficial effect of β-blockers includes resensitization and restoration of β1-adrenergic signaling pathways in the myocardium, improvements in cardiomyocyte contractility, and reversal of ventricular sensitization. However, emerging evidence indicates that another beneficial effect of β-blockers in disease may reside in sympathetic neurons. We investigated whether β-adrenoceptors are present on postganglionic sympathetic neurons and facilitate neurotransmission in a feed-forward manner. Using a combination of immunocytochemistry, RNA sequencing, Förster resonance energy transfer, and intracellular Ca^2+^ imaging, we demonstrate the presence of β-adrenoceptors on presynaptic sympathetic neurons in both human and rat stellate ganglia. In diseased neurons from the prehypertensive rat, there was enhanced β-adrenoceptor–mediated signaling predominantly via β_2_-adrenoceptor activation. Moreover, in human and rat neurons, we identified the presence of the epinephrine-synthesizing enzyme PNMT (phenylethanolamine-N-methyltransferase). Using high-pressure liquid chromatography with electrochemical detection, we measured greater epinephrine content and evoked release from the prehypertensive rat cardiac-stellate ganglia. We conclude that neurotransmitter switching resulting in enhanced epinephrine release, may provide presynaptic positive feedback on β-adrenoceptors to promote further release, that leads to greater postsynaptic excitability in disease, before increases in arterial blood pressure. Targeting neuronal β-adrenoceptor downstream signaling could provide therapeutic opportunity to minimize end-organ damage caused by sympathetic overactivity.

## Abstract 2

Supplemental Digital Content is available in the text.

The myocardial β-adrenergic receptor (βAR) signaling pathway plays a pivotal role in the pathogenesis of many cardiovascular diseases. Chronic cardiac adrenergic activation and impaired myocardial cyclic nucleotide (cN) signaling, resulting from enhanced catecholaminergic neurotransmission, are well-established contributors to ventricular hypertrophy, arrhythmia, and cardiomyocyte apoptosis.^1–3^ Sympathetic overactivity and vagal impairment (dysautonomia) are recurrent features in normotensive subjects with a familial predisposition for hypertension^4,5^ and in animal models of this disease.^6–8^ Moreover, patients with familial dysautonomia experience catecholaminergic supersensitivity, episodic hypertension, and have a high propensity for fatal cardiac events.^9^

β-Blockers are a mainstay treatment for many cardiovascular diseases and stress-related events.^10^ Chronic β-blocker therapy affords patients a wide-range of beneficial effects, including β_1_AR resensitization and restoration of intracellular cN signaling pathways, improvements in cardiac myocyte contractility, and reversal of ventricular remodeling.^1,3^ The precise mechanisms, however, that mediate and sustain the beneficial effects of β-blockers in disease remain unclear,^11,12^ although the presence of potentiating Gα_s_-coupled presynaptic βARs on presynaptic sympathetic terminals suggests a role for β-blockers in regulating cardiac-neuronal communication.^13–22^ The Adrenaline Hypothesis of hypertension argues that small incremental increases in plasma adrenaline (epinephrine) enhance sympathetic activity through sustained activation of presynaptic sympathetic βARs, leading to the development of hypertension.^17,23,24^ Whether epinephrine synthesis occurs before the onset of hypertension is not known, as there is limited cellular and molecular data within the sympathetic stellate ganglia to confirm this idea.

In this study, we investigated whether sympathetic βARs are present on human and rat sympathetic stellate ganglia (cervicothoracic ganglia, T1–T3) that preferentially innervate the heart.^25–28^ We aimed to establish whether intracellular second messenger signaling coupled to presynaptic βARs is impaired in prehypertensive states and contributes to altered Ca^2+^ and cN signaling before increases in arterial blood pressure. Finally, we aimed to assess which neurotransmitters are present within the cardiac-sympathetic ganglia to test the idea that epinephrine may act as the preferential sympathetic neurotransmitter, predisposing to disease.

## Methods

### Data Accessibility

Our RNA sequencing (RNAseq) raw FastQ files are deposited in the National Center for Biotechnology Information short reads archive under Short Reads Archive number SRP132271, and our quasi-mapped data will be available under Gene Expression Omnibus accession number (GSE110197).

### Clinical Samples

For clinical samples, human stellate ganglia were kindly sent by Drs Ajijola, Ardell, and Shivkumar from University of California, Los Angeles, Cardiac Arrhythmia Center. Characteristics of human donors are included in the online-only Data Supplement (Table S1 in the online-only Data Supplement). The human study was approved by the University of California, Los Angeles, Institutional Review Board (approval no. 12-000701), and informed consent was obtained from all subjects.

### Animals

Young male prehypertensive spontaneously hypertensive rats (pre-SHRs) of 3.5 to 5.5 week old, 16- to 20-week-old adult male normotensive Wistar rats, and age-matched spontaneously hypertensive rats (SHRs) were obtained from Envigo, United Kingdom. The SHR strain displays normal blood pressure at 4 weeks of age, where increases in arterial blood pressure develop progressively from 5 to 6 weeks of age.^29–36^ In this study, we used the Wistar rat strain as the normotensive control, given that Wistar rats are the progenitor strain from which the Wistar Kyoto was bred and the 2 strains display similar hemodynamic profiles at all ages.^31,32,36–41^ In addition, neither strain display a sympathetic Ca^2+^ phenotype (Figure S1A), making the Wistar a suitable control in this study. All rats were housed in standard plastic cages, and artificial lighting was fixed to a natural 12-hour light/dark cycle. Food and water were available ad libitum. All experiments were performed in accordance with the UK Home Office Animal Scientific Procedures Act 1986 and approved by the University of Oxford (PPL 30/3131; David J. Paterson). An expanded Materials and Methods section is available in the online-only Data Supplement for neuronal culture methodology, immunocytochemistry, RNAseq, Förster resonance energy transfer (FRET), Ca^2+^ imaging, and high-pressure liquid chromatography coupled to electrochemical detection protocols.

## Results

### Rat and Human Sympathetic Stellate Ganglia Express β_1_ and β_2_ Adrenoceptors

**Figure 1.**
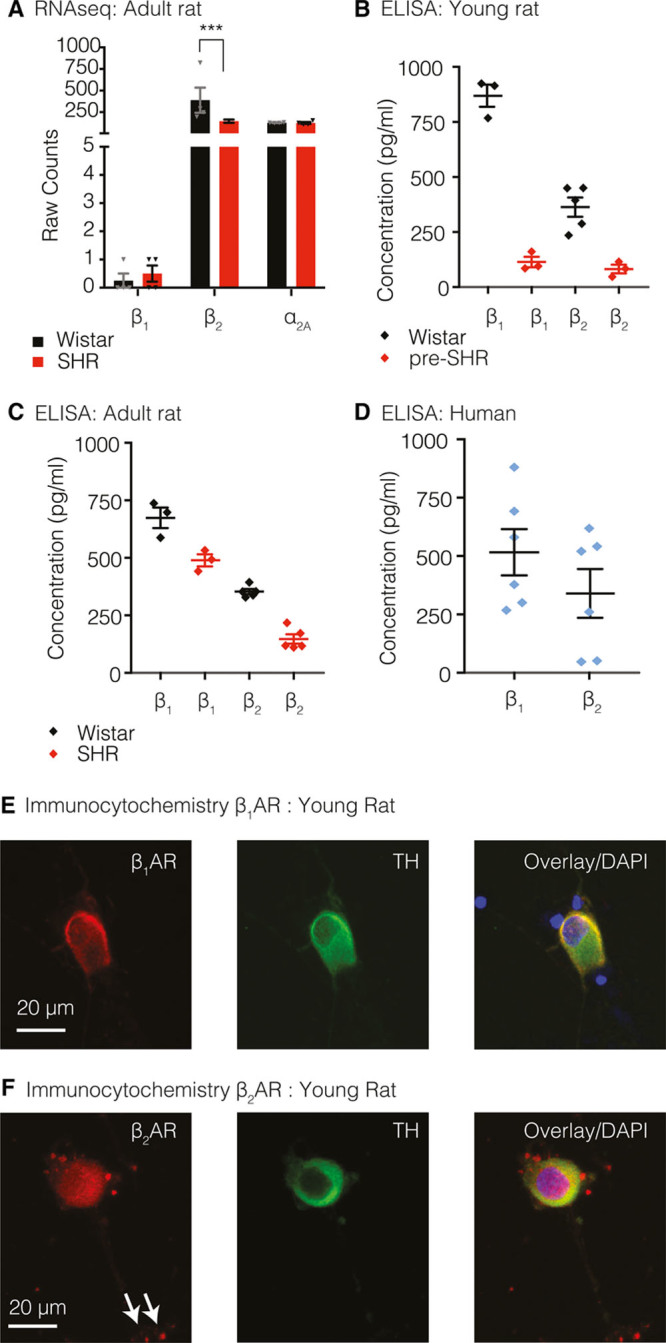
Rat sympathetic stellate ganglia express β_1_- and β_2_-adrenergic receptors (ARs). Using RNA sequencing, we identified the presence of β_1_AR (*Adrb1*), β_2_AR (*Adrb2*), and α_2A_AR (*Adra2a*) mRNA transcripts (**A**) from 16-wk-old male Wistar rats (n=4) and SHR (n=4). *Adrb2* expression was significantly lower in SHR ganglia compared with Wistar (*P*.adj=0.00945; Salmon-DESeq2 method). There was no significant difference in the levels of mRNA for *Adrb1* or *Adra2a* between strains or between age groups. Data points represent raw counts±SEM for each transcript (**A**). ELISAs confirmed protein expression of β_1_AR and β_2_ARs in 3.5- to 5-wk-old rat neurons (32 stellates, 16 animals/group) and 20-wk-old neurons (20 stellates, 10 animals/group); however, no statistical tests were conducted as stellates were pooled into a single sample to obtain adequate protein concentrations for the ELISA assays. In young rat stellates (**B**), the concentration of β_1_AR protein was calculated as 869.1±50.6 pg/mL (Wistar) and 114.2±23.7 pg/mL (pre-SHR). β_2_AR protein expression was calculated as 363.5±43.6 pg/mL (Wistar) and 82.2±20.0 pg/mL (pre-SHR). In adult rat stellates (**C**), β_1_AR expression was calculated as 674.1±44.6 pg/mL (Wistar) and 489.4±26.3 pg/mL (SHR). β_2_AR protein expression quantified as 353.3±11.2 pg/mL (Wistar) and 147.4±20.7 pg/mL (SHR). Data points depict mean±SEM of 3 to 4 technical replicates. β_1_AR (516±99.17 pg/mL) and β_2_AR (340±104.3 pg/mL) expression was also detected in stellate ganglia from human donors. Data points represent mean±SEM (6 replicates), from 3 pooled stellates obtained from 2 patients (**D**). Immunocytochemistry depicts β_1_AR (**E**) and β_2_AR (**F**) expression on TH (tyrosine hydroxylase)-positive neurons from 4-wk control rats. White arrows demonstrate the localization of β_2_AR on synaptic terminals.

We sequenced the transcriptome of the sympathetic stellate ganglia from 16-week-old male Wistar rats (n=4) and SHR (n=4). At 16 weeks, it is well-established that SHR display hypertension and sympathetic hyperactivity.^6,29–31,33,35,36,42^ Using quasi-mapping RNAseq^43^ and quantitative real-time (qRT)-polymerase chain reaction (PCR), we identified the presence of β_1_AR (*Adrb1*) and β_2_AR (*Adrb2*) mRNA transcripts, in addition to α_2A_AR (*Adra2a*) and tyrosine hydroxylase (*Th*) mRNA transcripts, markers of presynaptic sympathetic neurons, respectively (Figure 1A; Figure S1). We selected the α_2A_AR isoform as an indicator of presynaptic neuronal phenotype based on reports that the α_2A_AR primarily regulates presynaptic sympathetic activity.^44^ The α_2C_AR isoform plays a secondary role in regulating presynaptic norepinephrine release,^44^ whereas the α_2B_AR isoform has a preferential role within the vasculature.^44^ The mRNA expression for α_2A_AR was also found to be significantly higher than α_2C_AR expression identified by RNAseq (data not shown). Using RNAseq, we found that *Adrb2* mRNA expression was significantly lower in SHR ganglia compared with Wistar (Figure 1A; Figure S1C; *P*.adj=0.00945). Data points represent mean raw counts±SEM (Figure 1A).

The presence of *Adrb1, Adrb2, Adra2a*, and *Th* mRNA transcripts was identified and quantified by quantitative real time PCR (qRT-PCR) using RNA extracted from 4-week pre-SHR and Wistar rats (n=3 rats/group, unpooled; Figure S1D) and 16-week SHR and Wistar rats (n=4 rats/group, unpooled; Figure S1D). qRT-PCR data were analyzed using the ΔΔC_T_ method, where raw counts in both strains were first normalized to a control housekeeping gene *B2m*, and the difference in counts between SHR and Wistar was calculated.^45^ Data points represent log_2_ (fold change)±SEM. There was no significant difference in the levels of mRNA for *Adrb1*, *Adrb2, Adra2a*, or *Th* between strains or between age groups by qRT-PCR although the trend for a reduction in *Adrb2* expression remained.

Sandwich ELISAs were used to quantify the relative protein expression of β_1_AR and β_2_ARs in postganglionic sympathetic neurons obtained from 3- to 5-week-old normotensive pre-SHR and Wistar rats (Figure 1B; 32 stellates, 16 rats/group, pooled) or 19- to 20-week-old SHR and age-matched Wistar rats (Figure 1C; 20 stellates, 10 rats/group, pooled). The ELISA assays were biologically powered where 20 to 32 stellates were used per sample; however, the stellates tissue was pooled to obtain an adequate protein concentration for the ELISA assays, therefore no statistical comparisons were made. Data points indicate mean±SEM (of 3–4 technical replicates).

In 4 stellate ganglia samples obtained from 3 human donors (2 left stellates, 2 right stellates, unpooled), qRT-PCR confirmed the presence of mRNA transcripts encoding β_1_AR (*Adrb1*) and β_2_AR (*Adrb2*; S1E). Ganglia were α_2a_AR (*Adra2a*) positive, confirming a presynaptic phenotype. Samples were normalized to a control housekeeping gene *B2m* (3 replicates)using the ΔC_T_ method.^45^ Data points represent normalized counts±SEM. ELISAs confirmed the expression of β_1_AR (516±99.17 pg/mL) and β_2_AR (340±104.3 pg/mL) in 3 human stellates obtained from 2 patients (Figure 1D; pooled, 6 replicates). Immunocytochemistry confirmed the expression of β_1_AR and β_2_AR on TH-positive neurons from 3- to 5-week-old control rats (Figure 1E and 1F; respectively) and SHR (data not shown).

### βAR-Evoked cAMP Generation, PKA Activity, and [Ca^2+^]_i_ Are Enhanced in Pre-SHR Neurons

**Figure 2.**
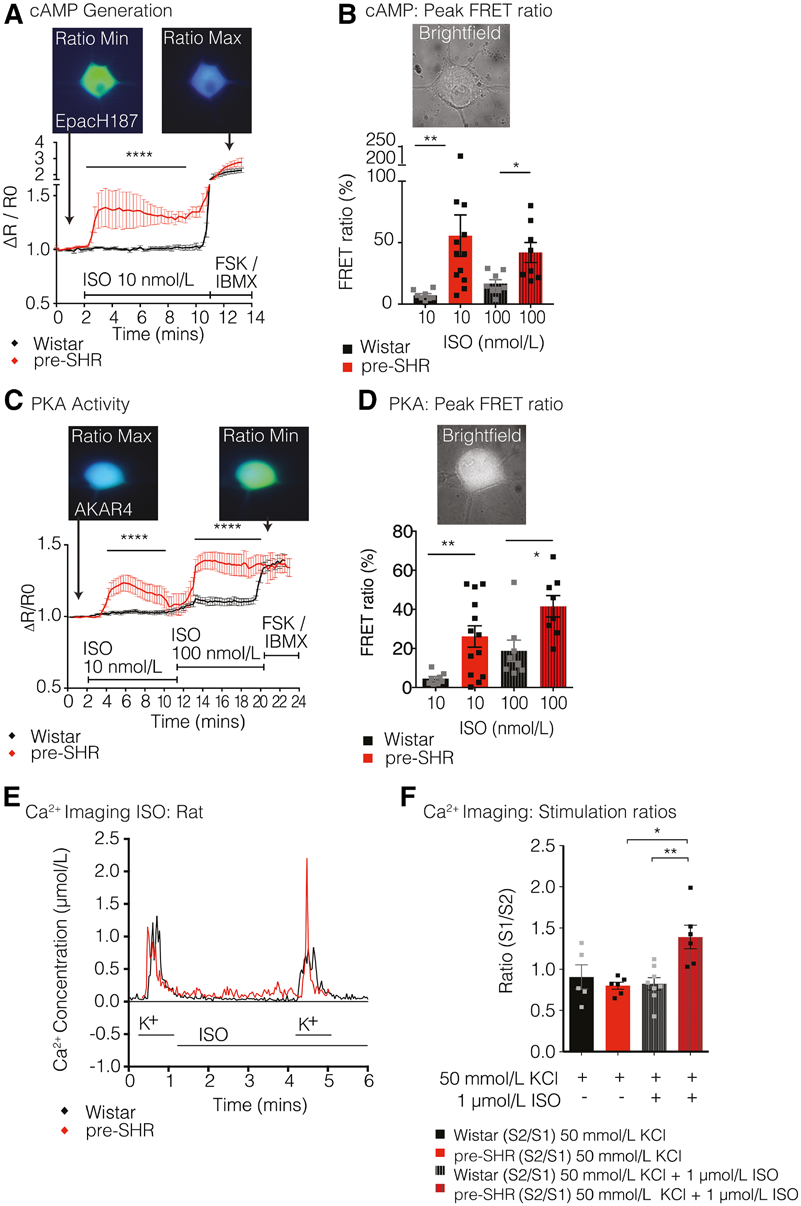
β-Adrenergic receptor (βAR) stimulation increases cAMP–PKA–Ca^2+^ signaling in pre-SHR neurons. Isoprenaline (ISO) generated significantly higher levels of cAMP at 10 nmol/L (**A**) in pre-SHR (55.6%±16.8%; n=12) compared with Wistar neurons (7.1%±1.4%; n=8; 2-way ANOVA; *P*<0.0001) and at 100 nmol/L (**B**) in pre-SHR (42.0%±8.2%; n=8) vs Wistar (16.8%±3.2%; n=8; 2-way ANOVA; *P*<0.0001). ISO increased PKA activity to a significantly greater extent at 10 nmol/L (**C**) in pre-SHR (26.1%±5.5%; n=13) vs Wistar neurons (4.5%±1.1%; n=8; 2-way ANOVA; *P*<0.0001) and at 100 nmol/L (**D**) in pre-SHR (41.5%±5.5%; n=8) vs Wistar (18.8%±5.4%; n=8; 2-way ANOVA; *P*<0.0001). Cells that did not respond appropriately to forskolin (FSK, 25 μmol/L) and IBMX (3-isobutyl-1-methylxanthine; 100 μmol/L) were excluded from the final analysis. Ca^2+^ imaging was conducted on neurons obtained from 4-wk rats using Indo-1AM (**E** and **F**). Wistar and pre-SHR neurons (n=8, 6, respectively) were exposed to 2 KCl challenges (50 mmol/L; stimulations 1 and 2) where stimulation 2 was conducted in the presence of ISO (1 μmol/L). Time-controlled experiments were performed in the absence of ISO (Wistar, n=5; and pre-SHR n=6). There was significantly higher [Ca^2+^]_i_ evoked in the presence of ISO in pre-SHR neurons compared with Wistar neurons (unpaired Student *t* test; *P*=0.0027). Bar charts represent mean±SEM. FRET indicates Förster resonance energy transfer.

To determine whether the presence of presynaptic βARs on sympathetic ganglia plays a functional role in modulating intracellular cN signaling pathways, we used FRET to quantify the relative levels of cAMP and PKA (protein kinase A) activity in response to a relatively nonselective βAR agonist, isoprenaline. To assess whether βAR-mediated cAMP generation facilitated signaling via the canonical cAMP–PKA–Ca^2+^ pathway, we used the loss-of-FRET sensor Epacs1-H187 (EpacH187)^46^ to measure changes in intracellular cAMP. Isoprenaline administration at 10 nmol/L led to significantly greater cAMP generation in pre-SHR (55.6%±16.8%) compared with that measured in Wistar neurons (7.1%±1.4%; 2-way ANOVA; *P*<0.0001) that was also observed at higher concentrations of isoprenaline (Figure 2A and 2B). PKA activity was measured using the gain-of-FRET sensor AKAR4.^47^ Using the same concentration of isoprenaline (10 nmol/L), we found that PKA activity was significantly higher in pre-SHR (26.1%±5.4%) versus Wistar neurons (4.5%±1.1%; 2-way ANOVA; *P*<0.0001), which was also observed at 100 nmol/L isoprenaline (Figure 2C and 2D). Raw YFP (yellow fluorescent protein) and CFP (cyan fluorescent protein) fluorescence traces as emitted from the cytosolic loss-of FRET sensor EpacH187 and the gain-of-FRET sensor AKAR4 in response to isoprenaline (10–100 nmol/L) are presented in the online-only Data Supplement (Figure S2A and S2B).

To assess whether isoprenaline-dependent βAR activation enhances intracellular Ca^2+^ ([Ca^2+^]_i_), we measured responses to KCl in the absence or presence of isoprenaline. Ca^2+^ recordings were obtained using Indo-1AM labeled sympathetic neurons from 4-week pre-SHR and Wistar rats. In pre-SHR stellate neurons, KCl stimulation in the presence of isoprenaline led to significantly higher [Ca^2+^]_i_ than KCl stimulations alone (Figure 2E and 2F; *P*=0.0272). There was significantly higher KCl-evoked [Ca^2+^]_i_ in the presence of isoprenaline in pre-SHR neurons compared with that recorded in control neurons (Figure 2E and 2F; *P*=0.0027). A time-controlled example trace is shown in the online-only Data Supplement (Figure S2C).

### Relative Contribution of β1AR and β2AR Signaling in Neuronal cAMP Generation

**Figure 3.**
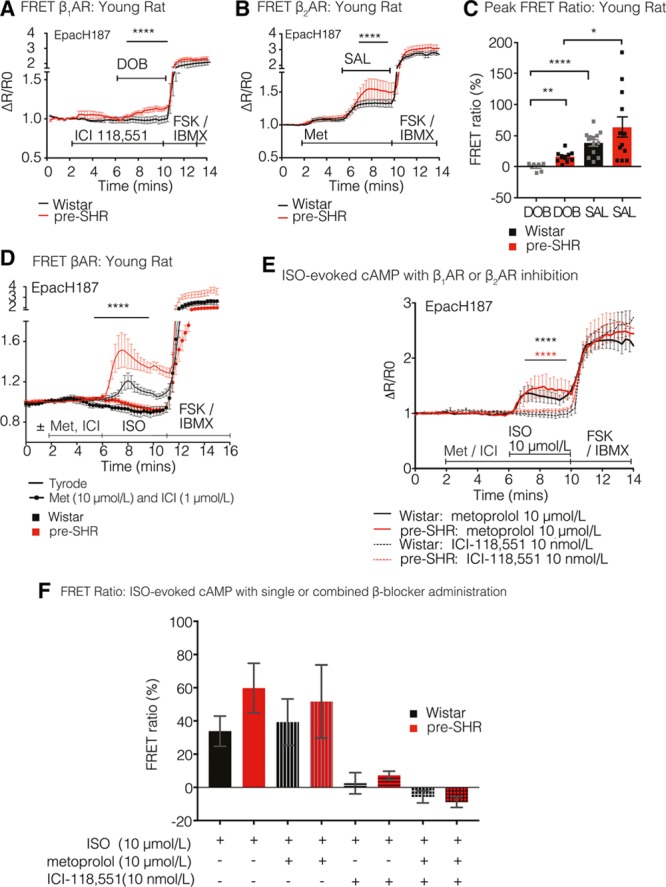
Relative contribution of presynaptic β_1_-adrenergic receptor (AR) and β_2_AR in neuron cAMP generation. For measurements of cAMP generation, 4-wk control and age-matched pre-SHR neurons were transduced with the EpacH187 FRET biosensor. Cells were stimulated with a β_1_AR agonist, dobutamine (DOB, 50 μmol/L) after β_2_AR inhibition with a selective antagonist, ICI-118,551 (ICI, 10 nmol/L) that led to a significantly greater increase in cAMP generation in pre-SHR (15.82%±2.8%; n=5) compared with Wistar neurons (−0.31%±2.4%; n=10; 2-way ANOVA; *P*<0.0001). In Wistar neurons, administration of DOB did not increase cAMP from baseline (**A**). In alterative experiments, neurons were stimulated with a β_2_AR agonist salbutamol (SAL, 10 μmol/L) after β_1_AR inhibition with a selective antagonist, metoprolol (MET, 100 nmol/L). SAL administration led to a greater increase in cAMP generation in pre-SHR (63.8%±16.6%; n=12) compared with Wistar neurons (38.6%±5.2%; n=13; 2-way ANOVA; *P*<0.0001; **B**). Peak FRET ratios (%) evoked by DOB or SAL were calculated (**C**). SAL generated significantly higher cAMP levels, than that evoked by DOB in Wistar (*P*<0.0001, Mann–Whitney) and in pre-SHR PGSNs (*P*=0.0173, unpaired 2-tailed Student *t* test). To confirm that ISO-evoked cAMP was acting downstream of βAR activation, we tested ISO-evoked cAMP in the absence and presence of a combination of β_1_AR and β_2_AR antagonists (ISO, 10 μmol/L; MET, 10 μmol/L, ICI, 1 μmol/L, respectively) or selective blockade of either β_1_AR (MET, 10 μmol/L) or β_2_AR (ICI, 10 nmol/L). The dual combination of β-blockers abolished cAMP generation in both pre-SHR (n=6) and Wistar neurons (n=6), demonstrating that ISO-dependent cAMP generation is dependent on βAR activation (**D**). There was significantly greater inhibition of cAMP after β_2_AR compared with β_1_AR inhibition in Wistar (*P*<0.0001; 2-way repeated measures ANOVA) and pre-SHR neurons (*P*<0.0001; 2-way repeated measures ANOVA), suggesting that β_2_AR plays a predominant role in cAMP generation, regardless of strain (**E**). Peak FRET responses are depicted (**F**).

To ascertain whether the observed increases in isoprenaline-evoked cAMP occurs predominantly through either β_1_AR or β_2_AR activation, cells were challenged with either a β_1_AR agonist (dobutamine, 50 μmol/L) after β_2_AR blockade with ICI-118,551 (ICI, 10 nmol/L) or in alternative experiments, administration of a β_2_AR agonist (salbutamol, 10 μmol/L) after β_1_AR antagonism (metoprolol, 100 nmol/L). Administration of the β_1_AR agonist dobutamine led to significantly greater cAMP generation in pre-SHR (15.82%±2.8%) compared with Wistar neurons (−0.31%±2.4%; 2-way ANOVA; *P*<0.0001; Figure 3A and 3C).

We observed that in Wistar neurons, administration of dobutamine did not increase cAMP from baseline. Administration of the β_2_AR agonist salbutamol also led to a significantly greater cAMP generation in pre-SHR (63.8%±16.6%) compared with Wistar neurons (38.6%±5.2%; 2-way ANOVA; *P*<0.0001; Figure 3B and 3C). There was significantly higher peak salbutamol-evoked cAMP compared with dobutamine-evoked cAMP in Wistar (*P*<0.0001; Mann–Whitney) and in pre-SHR neurons (*P*=0.0173, unpaired 2-tailed Student *t* test), highlighting a greater contribution of β_2_AR versus β_1_AR in generating cAMP, regardless of strain (C).

To confirm that isoprenaline-evoked cAMP is acting through β_1_AR and β_2_ARs rather than inducing off-target effects, we tested isoprenaline-evoked cAMP in the absence and presence of a combination of β_1_AR and β_2_AR antagonists (isoprenaline, 10 μmol/L; metoprolol, 10 μmol/L; ICI, 1 μmol/L, respectively). The combination of β_1_AR and β_2_AR antagonists abolished cAMP generation entirely in response to a high concentration of isoprenaline in both pre-SHR (n=6) and Wistar neurons (n=6), demonstrating that isoprenaline-dependent cAMP generation is dependent on selective β_1_AR and β_2_AR activation (Figure 3D). To support these observations, we also selectively inhibited β_1_AR (metoprolol, 10 μmol/L) or β_2_AR (ICI, 10 nmol/L) and measured the resulting cAMP generation in response to isoprenaline (10 μmol/L). β_2_AR blockade reduced isoprenaline-evoked cAMP generation to a greater extent than β_1_AR blockade in both Wistar and pre-SHR neurons, confirming our previous observations for a preferential effect of β_2_AR versus β_1_AR mediated signaling in postganglionic sympathetic neurons (Figure 3E and 3F). We measured a slight but significantly greater cAMP generation in pre-SHR versus Wistar neurons in the presence of either metoprolol (*P*=0.0472) or ICI (*P*<0.001) using 2-way repeated measure ANOVAs; however, the peak FRET responses themselves were not different significantly between strains (Figure 3F). The selectivity and specificity of the selected β_1_AR and β_2_AR agonists (dobutamine and salbutamol, respectively) and the β_1_AR and β_2_AR antagonists (metoprolol and ICI) have been previously reported.^48–51^

### Epinephrine-Synthesizing Enzyme PNMT Is Present in Rat and Human Stellate Ganglia

**Figure 4.**
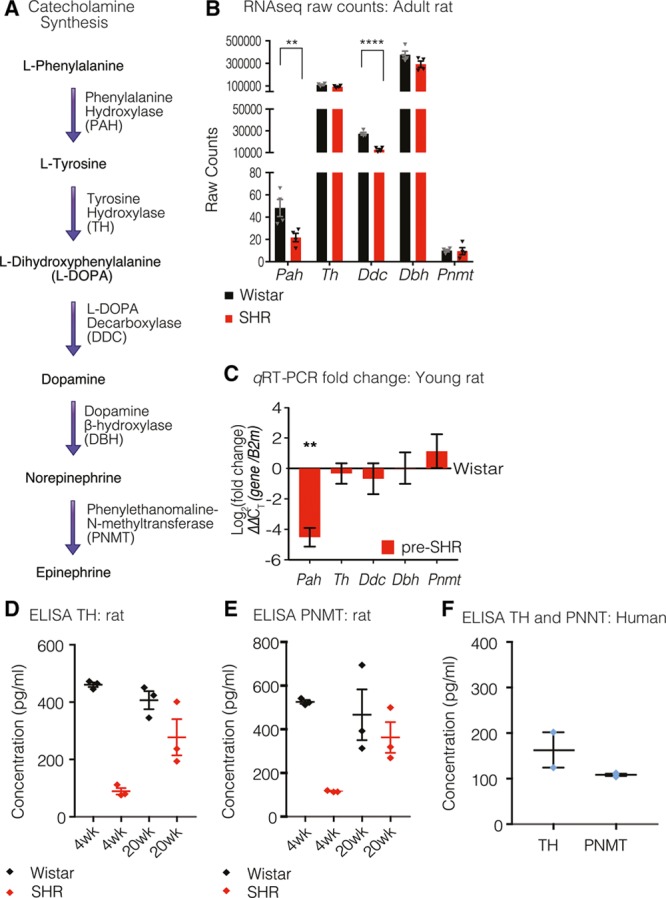
The epinephrine-synthesizing enzyme PNMT (phenylethanolamine-N-methyltransferase) is present in rat and human stellate ganglia. The catecholamine synthesis pathway is outlined (**A**). RNA sequencing (RNAseq) revealed mRNA transcripts that encode the enzymes required for norepinephrine (NE) synthesis: phenylalanine hydroxylase (*Pah*), tyrosine hydroxylase (*Th*), L-DOPA decarboxylase (*Ddc*), dopamine β-hydroxylase (*Dbh*; **B**). We also identified the transcript that encodes *Pnmt* required for the conversion of NE to epinephrine (Epi). *Pah* and *Ddc* mRNA expressions as determined by RNAseq were significantly lower in SHR neurons (*P.*adj=0.0719, *Pah*; 6.64×10^–15^, *Ddc*; Salmon-DESeq2). Data depicts raw counts±SEM (**B**). Transcript expression was validated via quantitative real-time polymerase chain reaction (qRT-PCR) using RNA extracted from 4-wk Wistar (n=4) and pre-SHR (n=4) ganglia (**C**). For qRT-PCR analyses, genes were normalized to the housekeeping gene (*B2m*), and SHR counts were normalized to Wistar using the ΔΔC_T_ method. There was a significant (4-fold) decrease in *Pah* in pre-SHR neurons (**C**; *P*=0.0098, unpaired 2-tailed Student *t* test). SHR (red bars) are depicted relative to number of counts calculated from Wistar samples (*x* axis). The protein concentration for TH (**D**) was quantified in 4-wk Wistar (460.9±7.979 pg/mL), pre-SHR ganglia (89.12±11.37 pg/mL), 20-wk Wistar (406.6±31.57 pg/mL), and SHR ganglia (277.5±63.03 pg/mL). PNMT protein expression was also quantified (**E**) in 4-wk Wistar (525.7±8.69 pg/mL), pre-SHR ganglia (117±3.73 pg/mL), 20-wk Wistar (466.7±116.2 pg/mL), and SHR ganglia (362.7±70.08 pg/mL). Data represent mean±SEM (2–3 technical replicates). We confirmed the protein expression of TH (163±38.83 pg/mL) and PNMT (108.4±2.386 pg/mL) in human stellates (**F**). Data points represent mean±SEM (2–3 replicates) from 3 pooled stellates obtained from 2 patients. Where stellates were pooled to obtain adequate protein concentrations, no statistical tests were conducted.

RNAseq was performed to obtain an overview of the transcriptome in stellate ganglia obtained from 16-week-old male SHR (n=4) and Wistar rats (n=4). We identified the presence of mRNA transcripts-encoding enzymes required for norepinephrine synthesis (Figure 4A): phenylalanine hydroxylase (*Pah*), *Th*, L-DOPA decarboxylase (*Ddc*), dopamine β-hydroxylase (*Dbh*). Furthermore, RNAseq identified the presence of the mRNA transcript encoding phenylethanolamine-N-methyltransferase (*Pnmt*), the enzyme required for the conversion of norepinephrine to epinephrine in both Wistar and SHR stellate ganglia. In the RNAseq data set, *Pah* and *Ddc* mRNA transcript expression were also shown to be significantly lower in SHR neurons (Figure 4B; Figure S3A; *P*.adj=0.0719; 6.64×10^–15^, *Pah*, *Ddc*, respectively). These findings were validated in 4-week Wistar and pre-SHR by qRT-PCR, and a significant ≈4-fold reduction in *Pah* expression was observed in pre-SHR ganglia (Figure 4C; *P*=0.0098). Data were normalized to a control housekeeping gene (*B2m*), and SHR gene counts were subsequently normalized to Wistar using the ΔΔC_T_ method. Data are presented as Log_2_(fold change).^45^ Using the same method, we also confirmed the presence of *Pnmt* and *Th* by qRT-PCR in neurons from 16-week Wistar and SHR (Figure S3B). There was no significant difference in mRNA expression of either *Th* or *Pnmt* between age groups or between phenotypes. ELISA assays confirmed protein expression of TH (Figure 4D) and PNMT (Figure 4E) in stellate ganglia from 4-week pre-SHR, 20-week-old SHR, and age-matched Wistar rats. The ELISA assays were high-powered biologically, where 20 to 32 stellates were used per sample; however, stellates were pooled to obtain adequate protein concentrations for the ELISA assays, therefore no statistical comparisons were made. Data points indicate mean±SEM (of 2–3 technical replicates).

To assess whether the presence of PNMT in sympathetic stellate ganglia is conserved in higher species, we obtained stellate ganglia from male human donors. qRT-PCR demonstrated the presence of both *Th* and *Pnmt* mRNA transcripts in human sympathetic stellate ganglia (Figure S3C). Data were normalized to a control housekeeping gene *B2m* using the ΔC_T_ method^45^ and expressed as normalized count values (3 patients, 4 stellates). We also used ELISAs to confirm protein expression of both TH and PNMT in human stellate samples (Figure 4F). Data points represent mean±SEM (2–3 replicates) from 3 pooled stellates obtained from 2 patients.

### Epinephrine Is Released From Pre-SHR but Not Wistar Whole-Stellate Ganglia

**Figure 5:**
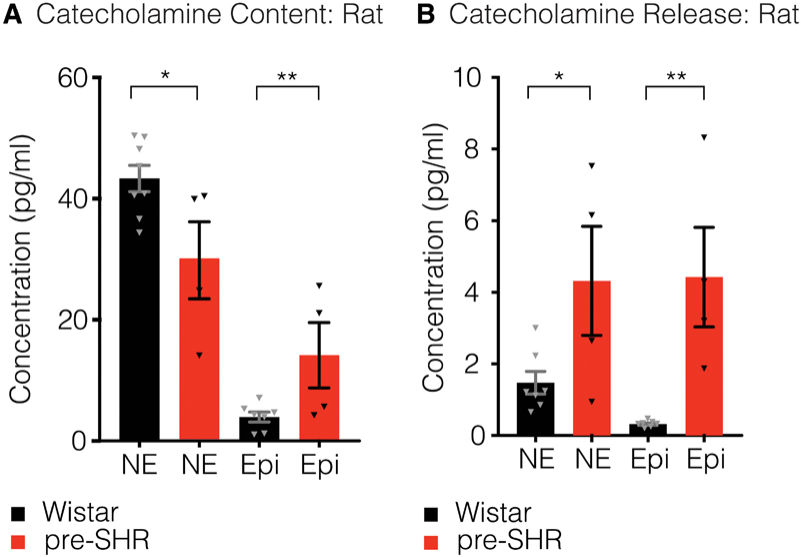
. Epinephrine (Epi) is released from pre-SHR but not Wistar whole-stellate ganglia. Using high-pressure liquid chromatography coupled to electrochemical detection, we measured significantly higher total norepinephrine (NE; **A**) in Wistar (43.3±2.173 pg; n=8) compared with pre-SHR neurons (29.82±6.366 pg; n=4; unpaired 2-tailed Student *t* test; *P*=0.0294). In the same stellate samples (**A**), we also measured a significantly greater total content of Epi in pre-SHR (14.14±5.399 pg) compared with that measured in Wistar ganglia (3.937±0.820 pg; unpaired 2-tailed Student *t* test; *P*=0.0019). Electric field stimulation of whole-rat stellate ganglia led to the release of NE (**B**) that was significantly higher in samples obtained from pre-SHR (4.32±1.523 pg) vs Wistar ganglia (1.477±0.316 pg; unpaired 2-tailed Student *t* test; *P*=0.0396). The concentrations of neurally-mediated Epi release (**B**) were also significantly higher in pre-SHR (4.424±1.391 pg; n=4) compared with Wistar stellates (0.3201±0.0325 pg; n=8; unpaired 2-tailed Student *t* test; *P*=0.0028).

After the identification of PNMT, we investigated whether epinephrine is released from the whole rat stellate ganglia under basal conditions or with electric field stimulation. We measured significantly greater total norepinephrine content in homogenized Wistar stellates (43.3±2.173 pg; n=8) compared with pre-SHR stellate ganglia (29.82±6.366 pg; n=4; *P*=0.0294). In the same homogenate, we measured a greater content of epinephrine in pre-SHR ganglia (14.14±5.399 pg) compared with that measured in Wistar stellates (3.937±0.820 pg; *P*=0.0019), suggesting that a significant amount of norepinephrine is converted to epinephrine in prehypertensive states (Figure 5A).

We also investigated whether epinephrine is released from rat stellate ganglia with electric stimulation (Figure 5B). Electrically evoked concentrations of norepinephrine were significantly higher in samples obtained from pre-SHR (4.32±1.523 pg; n=4) versus Wistar ganglia (1.477±0.316 pg; n=8; *P*=0.0396). Moreover, in the same samples, the concentrations of electrically evoked epinephrine were significantly higher in pre-SHR ganglia (4.424±1.391 pg) compared with that measured in Wistar stellates (0.3201±0.0325 pg; *P*=0.0396).

## Discussion

In this study, we have obtained evidence for β_1_AR and β_2_AR mRNA and protein expression on presynaptic postganglionic sympathetic neurons from human and rat ganglia. We have further demonstrated that in isolated sympathetic neurons, βAR agonists elevate cAMP and activate PKA. The effects were more pronounced in neurons from pre-SHR rats. We also observed that βAR agonists enhanced [Ca^2+^]_i_in response to depolarization by high K^+^ in pre-SHR neurons only. In addition, we demonstrate the presence of mRNA and protein expression of PNMT, the enzyme involved in the synthesis of epinephrine in human and rat sympathetic stellate neurons. Moreover, we observed that epinephrine is present in diseased states and is actively released from prehypertensive, but not healthy rat neurons, suggesting preferential switching of neurotransmitter synthesis in disease.

Single or combinatorial administration of β-blockers is a mainstay treatment strategy for diseases caused by sympathetic overactivity, although the precise mechanisms that underpin the long-term beneficial effects are not entirely clear.^12^ Current dogma suggests that the observed antihypertensive and cardioprotective effects of β-blockers are mediated through inhibition of cardiac and vascular βARs, reducing myocardial work and total peripheral resistance.^52^ Our findings suggest that the efficacy of clinical β-blockers may be attributed, at least in part, to a reduction in sympathetic hyperactivity and neurotransmission at the end-organ.

What is the cause for increased sympathetic neurotransmission before the onset of neurogenic hypertension? Emerging evidence suggests that impaired nitric oxide synthesis and reductions in cGMP–PKG (protein kinase G) signaling lead to pathological increases in [Ca^2+^]_i_ and norepinephrine release at the end-organ.^31,53^ Recently, we demonstrated that decreased cGMP signaling leads to enhanced N-type Ca^2+^ channel (Ca_v_2.2) currents and that this effect may be ameliorated by artificially increasing cytosolic cGMP.^54,55^ cN signaling is acutely regulated by phosphodiesterase enzymes, and in early prehypertensive states, phosphodiesterase signaling is impaired, resulting in an imbalance between cAMP and cGMP signaling.^54^ We, therefore, sought to ask the question, could high levels of neurotransmitter release act in an autocrine or paracrine fashion to increase neuronal cAMP and potentiate neurotransmission in a feed-forward manner?

Although it has been previously reported that presynaptic βARs are present^56^ and may be capable of facilitating norepinephrine release in several peripheral autonomic ganglia in rat, guinea pig, cat, rabbit, dog, and human^13–17,19,57–62^, the role of adrenergic signaling within the sympathetic stellate ganglia remains unclear, particularly in disease. In the present study, we confirmed the presence of both β_1_AR and β_2_AR isoforms in stellate ganglia from human and rat and found that activation of βARs on rat sympathetic neuron led to a significantly greater increase in intracellular cAMP generation, PKA activity in pre-SHR compared with control neurons (Figure 2).

To assess whether βAR signaling facilitates cardiac-sympathetic neurotransmission, [Ca^2+^]_i_ was measured in response to KCl in the absence or presence of isoprenaline. Consistent with the observed increases in βAR-mediated cAMP–PKA signaling in pre-SHR neurons, isoprenaline also increased KCl-evoked [Ca^2+^]_i_ in prehypertensive states; whereas there was no effect of isoprenaline in control neurons (Figure 2E and 2F). These data demonstrate that enhanced βAR-mediated signaling in sympathetic neurons contributes to the Ca^2+^ phenotype and increases sympathetic transmission. Previous work has demonstrated that the N-type calcium channel is the primary voltage-gated channel responsible for Ca^2+^ influx in sympathetic neurons and carries a significantly larger Ca^2+^ current in pre-SHR and SHR neurons compared with controls.^55,63^ N-type calcium channelactivity is differentially regulated by PKA and PKG.^54,55^ Therefore, we suggest that the isoprenaline-potentiated increases in [Ca^2+^]_i_ in pre-SHR neurons primarily occurs as a result of βAR–cAMP activation that increases PKA-dependent phosphorylation of N-type calcium channel (Ca_v_2.2).

To establish whether the observed increases in cAMP–PKA activity occur downstream of β_1_AR or β_2_AR signaling, cells were perfused with selective agonists for either β_1_AR or β_2_AR subtypes in the presence of either alternate βAR antagonist. We found that selective activation of β_1_AR or β_2_AR led to significantly greater increases in cAMP in pre-SHR neurons compared with Wistar. Indeed, there was no measureable effect of β_1_AR activation on [cAMP] in normotensive controls (Figure 3). Furthermore, stimulation of pre-SHR neurons with the β_2_AR agonist salbutamol led to cAMP generation that was almost twice as high as β_1_AR-evoked cAMP within pre-SHR neurons, suggesting a dominant role for β_2_AR compared with β_1_AR signaling. To establish whether increased βAR signaling in pre-SHR results from increases in βAR expression, we measured levels of β_1_AR and β_2_AR mRNA via qRT-PCR and RNAseq and quantified protein levels using sandwich ELISAs. Surprisingly, we observed that βAR transcripts and protein expression are reduced in pre-SHR stellates, as well as in aged SHR with established hypertension,^6,29–31,33,35,36,42^ compared with age-matched Wistar neurons, in a similar manner to that reported in the myocardium.^64,65^ We also report that in healthy ganglia, β_1_AR expression decreases with age, much like in the heart (Figure 1). Together, these data suggest that in diseased states, the potentiating effects of βAR agonists may be mediated through impaired second messengers coupled to cAMP and its effector PKA, probably via impairment of phosphodiesterases to hydrolyze cAMP^54,55^ rather than the G-protein coupled receptors themselves.

Which neurotransmitter preferentially activates presynaptic βARs? Several studies suggest limited involvement of norepinephrine in potentiating presynaptic neurotransmission but argue for a critical role for epinephrine in enhancing release, particularly in patients with essential hypertension^66^ or stress disorders.^67,68^ The role of epinephrine in the pathogenesis of essential hypertension has been termed the Adrenaline Hypothesis^23^; however, the origins of local concentration of epinephrine remain unclear. Most reports suggest that high circulating plasma epinephrine concentrations arise from the adrenal medulla with active reuptake into sympathetic nerve terminals.^19,22,23,69–71^ Others have identified heightened epinephrine synthesis within the central nervous system, specifically the nucleus tractus solitariuus^72^ and hypothalamus^72,73^ and suggest that this source of epinephrine may underpin the high plasma levels of epinephrine. Alternatively, some reports have identified in situ epinephrine synthesis within various sympathetic ganglia in rat and human, via a stress-inducible mechanism.^19,24,66,72,74–77^ Our identification of PNMT mRNA and protein expression in human and rat cardiac-sympathetic ganglia (Figure 4) supports the findings of these earlier studies that epinephrine is synthesized in sympathetic stellate ganglia in disease.

What is the relevance of epinephrine synthesis in prehypertensive sympathetic stellate ganglia? The Adrenaline Hypothesis of hypertension proposes that stress and subsequent small incremental increases in epinephrine plays a major role in the pathogenesis of hypertension, not via epinephrine directly, but as a result of increased sympathetic activity and enhanced norepinephrine release.^23^ This sustained increase in sympathetic activity caused by epinephrine leads to the development of hypertension. We have shown that epinephrine is synthesized in pre-SHR to a greater extent than in healthy sympathetic stellate ganglia and is only released from pre-SHR ganglia. Importantly, epinephrine has a 10-fold higher affinity for β_2_AR than norepinephrine (EC_50_ 5.2, 53.7 nmol/L, respectively) and is capable of generating 3× more cAMP than norepinephrine via β_2_AR activation, a feature that may be mimicked by isoprenaline because of similarities in efficacy.^78^ Subsequently, the high efficacy of epinephrine (and the relatively low efficacy of norepinephrine) at β_2_AR has been shown to result in epinephrine-dependent norepinephrine transmission. Indeed, low concentrations of epinephrine (0.1–10 nmol/L) have been shown to be 100× to 500× more potent than norepinephrine in enhancing activity-dependent norepinephrine release.^19,79–81^ Moreover, epinephrine may have a more sustained effect on norepinephrine release because of the extended tissue half-life of epinephrine.^79^ Epinephrine-induced norepinephrine release has been identified in a wide variety of peripheral tissues in rat, rabbit, and human.^18,19,22^

We sought to investigate whether the presence of presynaptic PNMT plays a functional role in converting norepinephrine to epinephrine in rat stellate ganglia, by measuring total catecholamine content and electrically evoked catecholamines by high-pressure liquid chromatography coupled to electrochemical detection (Figure 5). We identified a significant decrease in total norepinephrine content in pre-SHR ganglia (74.8% of total catecholamine content) compared with norepinephrine calculated in Wistar ganglia (91.5% of total catecholamine content). We have also observed that the total content of epinephrine was significantly higher in pre-SHR ganglia (25.2% of total catecholamine content) compared with epinephrine levels quantified in Wistar ganglia (8.5% of total catecholamines measured). Furthermore, we found that on electric stimulation, the percentage ratio of norepinephrine:epinephrine released from Wistar ganglia was calculated as 91%:9%; whereas in pre-SHR ganglia, the ratio of catecholamines released (norepinephrine:epinephrine) was 44%:56% (Figure 5), although the total amounts of catecholamines released during electric stimulation remained fairly similar between the strains (≈11–12 pg).

One recurrent feature in human and animal models of hypertension is the reduction in norepinephrine reuptake transport (NET), leading to larger and more sustained extracellular catecholamine concentrations.^24,82–84^ Recently, it has been proposed that PNMT may also act as a DNA methylase, silencing NET transcription that may underpin the observed NET phenotype.^24^ We have previously identified reductions in NET activity in the pre-SHR cardiac-stellate ganglia.^82^

### Limitations

In this study, we investigated the role and mechanisms involved in feed-forward presynaptic signaling in the cardiac-sympathetic ganglia. We performed a hypothesis neutral, nonbiased approach to sequencing the transcriptome of sympathetic stellate in adult rats that revealed the presence of RNA transcripts involved βAR receptor expression and epinephrine synthesis. We assessed the functional relevance of these findings by probing the adrenergic intracellular signaling pathways coupled to Ca^2+^-mediated exocytosis. There were several limitations to these approaches. First, the stellate ganglion comprises a heterogeneous population of cell types. Indeed, we identified markers of fibroblasts and astrocytes, including vimentin and glial fibrillary acidic protein, respectively; however, we identified that a high number of transcripts were neuronal in phenotype. We also found that the subunit profile of nicotinic acetylcholine receptors matches those described for sympathetic neurons.^85^ Moreover, immunocytochemistry highlighted the localization of β- and αARs on the soma and dendrites of TH-positive neurons. In support of these data, our collaborators have also identified the presence of transcripts encoding presynaptic βARs in sorted sympathetic mouse neurons (Ana Domingos, personal communication, 2018). Second, in the absence of cardiac tracing experiments, we rely on anatomic literature,^25–27^ and our own previous observations^32,86–88^ that the results presented here are relevant to cardiac-sympathetic communication because significant myocardial sympathetic innervation has been shown to arise from the cervicothoracic ganglia. Third, we used stellates obtained from male rats. Although sex differences in hypertension and cardiovascular disease incidence have been widely reported,^89^ in this study, we focused on investigating the transcriptome of the male rat stellate ganglia given that the prevalence for cardiovascular diseases is significantly higher in males than premenopausal women.^89^ Fourth, cNs and phosphodiesterases reside in distinct subcellular compartments, and their localization with βARs receptors is acutely regulated.^90–92^ Similarly, the regulation of Ca^2+^ channels by PKA/PKG occurs in distinct signalosomes, conferring site-specific regulation of Ca^2+^ entry coupled to neurotransmission. Furthermore, the rate of phosphodiesterase hydrolysis is critically dependent on the concentration of both cAMP and cGMP that is reported to be different between cell types.^93^ In this study, we measured global cytosolic cAMP, PKA, and Ca^2+^ concentrations, therefore we cannot ascertain precisely where the key pathways converge. Site-specific FRET and Ca^2+^ sensors will be required to resolve the question of microdomain impairments in cN and effector signaling.

### Perspectives

**Figure 6.**
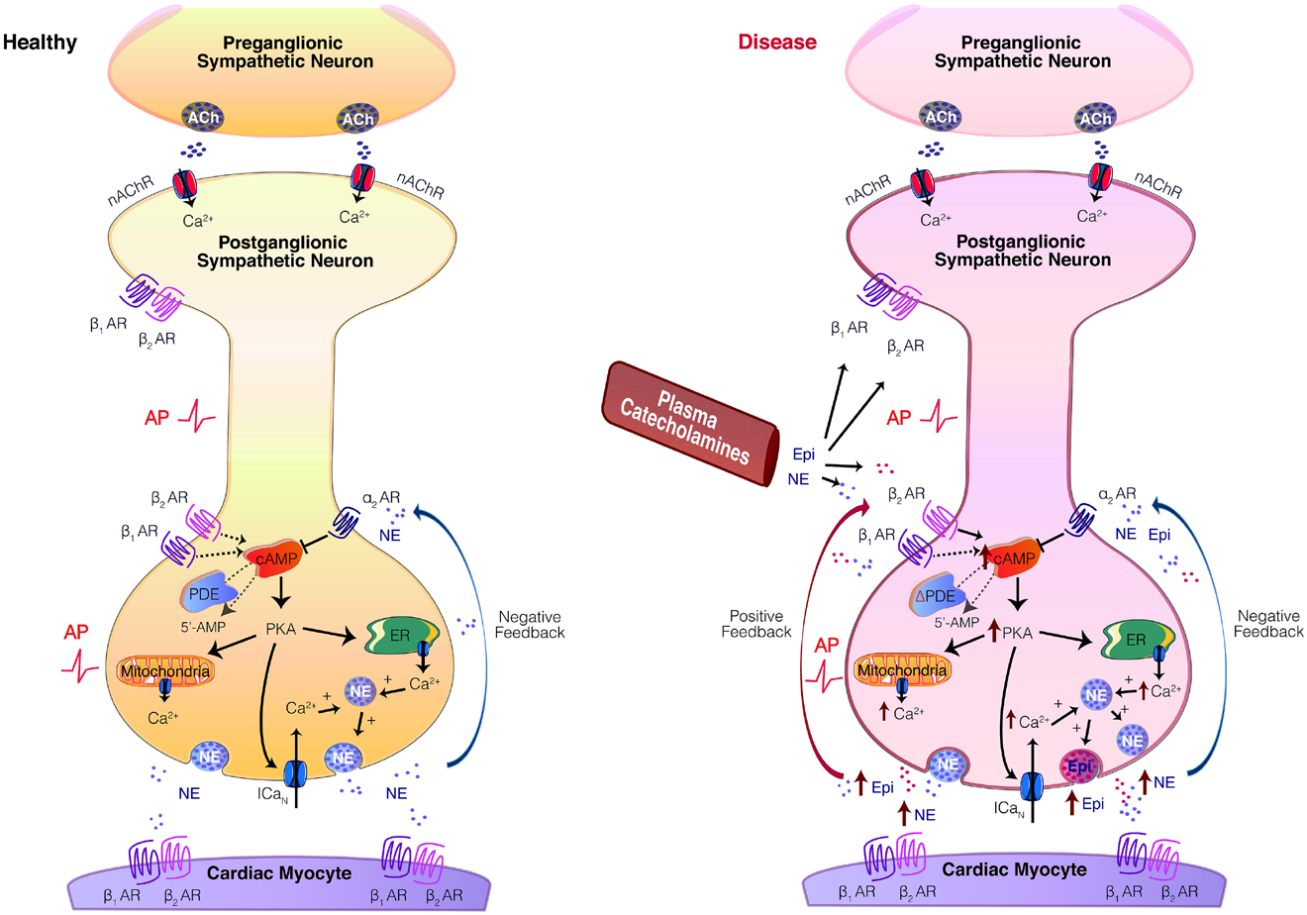
Model figure. The sympathetic stellate ganglia (cervicothoracic ganglia) are located alongside vertebrate T1 to T3. They are the primary sympathetic ganglia that innervate the heart and have been shown to exert the greatest control over increases in heart rate and contractility.^25–28^ In healthy postganglionic sympathetic neurons (**A**), Ca^2+^-dependent exocytosis facilitates the release of norepinephrine (NE) onto cardiac myocytes, where postsynaptic β_1_- and β_2_-adrenergic receptors (ARs) are activated. Increases in extracellular NE acts on presynaptic α_2_ARs, reducing adenylyl cyclase (AC) activity through activation of inhibitory Gαi G-proteins. Acute regulation of cAMP is maintained by phosphodiesterases (PDEs).^90,91^ cAMP-dependent PKA (protein kinase A) activity increases intracellular Ca^2+^ ([Ca^2+^]_i_) via phosphorylation of the N-type Ca^2+^ Channel (ICaN; CaV2.2)^55^; regulation of endoplasmic reticulum stores and mitochondrial Ca^2+^ release.^31^ In neurons obtained from the prehypertensive SHR, a young genetic model of hypertension (**B**), Ca^2+^-dependent exocytosis facilitates the release of NE and epinephrine (Epi). Activation of presynaptic βARs in prehypertensive states^29–36^ enhances cAMP generation, PKA activity, and [Ca^2+^]_i_ to greater levels than in healthy neurons, facilitating neurotransmission in a potentiating feed-forward manner. This occurs preferentially via β_2_AR activation. Catecholamines may also be supplied from the circulation. We propose that β-blockers may have efficacy at βARs expressed on peripheral neurons, by reducing cardiac-sympathetic communication in hypertension and dysautonomias. ACh indicates acetylcholine; and nAChRs, nicotinic acetylcholine receptors.

Our data here demonstrate that in prehypertensive and hypertensive states, epinephrine is synthesized within presynaptic sympathetic nerve terminals and released on activation at the end-organ. In prehypertensive states, evoked release of epinephrine (that is exacerbated by decreased NET activity) may act preferentially on presynaptic β_2_ARs to increase cAMP generation and PKA activity, thereby enhancing Ca^2+^ levels and neurotransmission in disease, in a manner akin to positive feedback. We suggest that epinephrine release at the end-organ may play a role in the pathogenesis of hypertension. Figure 6 depicts the model signaling pathways in healthy and prehypertensive states and highlights potential sites for neural phenotypic targeting in disease. We suggest that in a model of early hypertension, activation of presynaptic βARs enhances cAMP generation, PKA activity, and [Ca^2+^]_i_ to greater levels than that measured in healthy neurons, facilitating both norepinephrine and epinephrine release. Presynaptic activation of β_2_ARs (and β_1_ARs to a lesser extent) further enhances neurotransmission in a potentiating feed-forward manner, with activation of α_2_ARs playing a role in negative feedback. Additional studies aimed at investigating the relative roles of epinephrine and norepinephrine in positive and negative feedbacks signaling may be of therapeutic relevance. Indeed, these findings may have implications beyond neurogenic hypertension and may offer benefit in other diseases of sympathetic overactivity, such as modulation of renin–angiotensin–aldosterone release, chronic inflammatory diseases, and heart failure.

## Acknowledgments

We acknowledge the High-Throughput Genomics Group at the Wellcome Trust Centre for Human Genetics (funded by Wellcome Trust grant reference 090532/Z/09/Z) for the generation of the sequencing data. We acknowledge our collaborators Drs Ajijola, Shivkumar, and Ardell at the University of California, Los Angeles, Cardiac Arrhythmia Center for kindly extracting and shipping human sympathetic stellate ganglia from donor patients no. 19, no. 23, and no. 24. We thank Dr Threlfell in our department for her help and expertise with high-pressure liquid chromatography coupled to electrochemical detection experimental design and protocols. E.N. Bardsley and D.J. Paterson planned the project. E.N. Bardsley performed all of the rat and human RNA extraction, validation, and quantitative real-time polymerase chain reaction experiments. E.N. Bardsley performed all cell culturing and performed Ca^2+^ imaging, Förster resonance energy transfer imaging, ELISAs, and immunocytochemistry. K.J. Buckler assisted with the Ca^2+^ experiments. H. Davis performed the catecholamine high-pressure liquid chromatography coupled to electrochemical detection experiments. E.N. Bardsley analyzed all experimental data. E.N. Bardsley and H. Davis performed the RNA sequencing differential expression analysis. E.N. Bardsley and D.J. Paterson cowrote the paper. All authors edited the manuscript.

## Sources of Funding

This project was funded by the Wellcome Trust OXION initiative (105409/Z/14/Z), British Heart Foundation (BHF) Centre of Research Excellence and BHF (RG/17/14/33085), and National Institutes of Health SPARC (OT2OD023848) initiative.

## Disclosures

None.

## Supplementary Material

## Novelty and Significance

### What Is New?

- We provide evidence for β_1_- and β_2_-adrenergic receptors in human stellate ganglia. These receptors are also conserved in rat stellates. We demonstrate that the cAMP–PKA signaling pathway coupled to activation of β-adrenergic receptors is impaired and exacerbates the Ca^2+^ phenotype in young prehypertensive rats.
- We have also shown that prehypertensive rat stellate neurons synthesize and release epinephrine.

### What Is Relevant?

- Neurotransmitter switching to epinephrine may be an early cellular marker because this neurochemical phenotype may reflect sympathetic impairment in the early stages of disease progression.
- Targeting the β-adrenergic intracellular signaling pathway and reducing PNMT (phenylethanolamine-N-methyltransferase) activity may be therapeutically relevant for the early treatment of sympathetic dysautonomia.

### Summary

Using a combination of RNA sequencing, quantitative real-time polymerase chain reaction, immunocytochemistry, ELISA, Förster resonance energy transfer and Ca^2+^ imaging, we have shown the presence β-adrenergic receptors on presynaptic sympathetic neurons in human and rat. We have demonstrated that catecholaminergic stimulation of these receptors exacerbates the sympathetic Ca^2+^ phenotype in the pre-SHR before the onset of hypertension. Using high-pressure liquid chromatography coupled to electrochemical detection, we have also found that physiological concentrations of epinephrine are released from sympathetic ganglia before increases in blood pressure occur. This may reflect a site of neural impairment in disease progression.
