# Supplementary material for: Neurotransmitter Switching Coupled to β-Adrenergic Signaling in Sympathetic Neurons in Prehypertensive States
Source: Hypertension. 2018 May 9;71(6):1226–38. doi: 10.1161/HYPERTENSIONAHA.118.10844 (PMC5959210; doi:10.1161/HYPERTENSIONAHA.118.10844)
Supplement: Supplementary file 1 [file hyp-71-1226-s001.docx]

**ONLINE SUPPLEMENT**

**NEUROTRANSMITTER SWITCHING COUPLED TO BETA-ADRENERGIC SIGNALING IN SYMPATHETIC NEURONS IN PREHYPERTENSIVE STATES**

Emma N. Bardsley*^1^, Harvey Davis^1^, Keith J. Buckler^1^, David J. Paterson*^1^

^1^Wellcome Trust OXION Initiative in Ion Channels and Disease, Burdon Sanderson Cardiac Science Centre, Department of Physiology, Anatomy and Genetics, Sherrington Building, University of Oxford, Oxford, OX1 3PT, UK

***Corresponding Authors:** Miss Emma Nicole Bardsley, University of Oxford, Department of Physiology, Anatomy and Genetics, Oxford, OX1 3PT, UK; [emma.bardsley@dpag.ox.ac.uk](mailto:emma.bardsley@dpag.ox.ac.uk), tel. +44 (0)1865 272471.

Prof. David James Paterson, University of Oxford, Department of Physiology, Anatomy and Genetics, Oxford, OX1 3PT, UK; david.paterson@dpag.ox.ac.uk, tel. +44 (0)1865 272471.

**Short title:** Neurotransmitter Switch in Prehypertension (42 characters)

**Supplemental Materials and Methods**

***Reagents.*** All drugs and reagents were sourced from Sigma-Aldrich UK unless otherwise stated.

***Adenovirus generation.*** The cAMP-sensitive FRET sensor Epacs1-H187 (EpacH187) and the PKA-sensitive FRET sensor AKAR4 were obtained as described previously^1^. Generation and amplification of the virus particles was outsourced to Vector Biolabs.

***Neuronal Isolation from Rat Sympathetic Cardiac Ganglia.*** Rats were anaesthetized in an induction chamber (3-5% isoflurane) and humanely killed by a Home Office approved Schedule 1 method: overdose of pentobarbital (Euthatal, 200 mg/mL) and exsanguination. The stellate ganglia were removed, de-sheathed from connective tissue, dissected and enzymatically digested with collagenase (type IV, 1 mg/mL, 37 °C) and trypsin (type I, 1 mg/mL, 37 °C). Stellates were washed with a modified L-15 blocking medium that was equilibrated prior to use (37 °C, 5 % CO_2_) and the ganglia were dissociated into a single-cell suspension via manual trituration in pre-equilibrated plating medium (37 °C, 5 % CO_2_). Post-ganglionic sympathetic neurons (PGSNs) were plated on Poly-D-Lysine/laminin-coated coverslips (6 mm, VWR International) and incubated in plating medium (37 °C, 5 % CO_2_) until required for use.

***Immunocytochemistry.*** PGSNs were cultured from 3-5-week preSHR and Wistar control rats. Stellate neurons were fixed with paraformaldehyde (2%, 5 mins) before blocking and permeablizing with goat serum (10%), bovine serum albumin (0.3%) and Triton X-100 (0.1%) in dPBS without Ca^2+^ and Mg^2+^ (1-hour, room temperature (RT)). Cells were washed thoroughly with dPBS (3 x 10 min) and incubated in primary antibodies (1-hour, 37 °C). The following antibodies were used: anti-tyrosine hydroxylase (TH, 1:250, T1299, Abcam), anti-β_1_AR (ab3442, 1:100, Abcam), anti-β_2_AR, (ab182136, 1:200, Abcam). Cells were incubated with the appropriate secondary antibodies (Alexa Fluor, 1:1000, 2 hours, 37 °C) before they were mounted on microscope slides using medium containing DAPI (Vectashield, Vector Laboratories) and sealed. Control experiments that were carried out in the absence of primary antibodies did not display any fluorescence except DAPI, confirming the absence of non-specific binding. Specificity of primary antibodies for β_1_AR and β_2_AR was also validated via Western blotting (data not shown). Cells were imaged using 40X or 60X objectives on a confocal microscope (Live Cell Olympus Inverted Confocal or Zeiss LSM 880 Airy Scan Upright Confocal). Z-stack images were edited and cell sizes were measured using Fiji software (ImageJ)^2,3^.

***RNA Extraction from Sympathetic Cardiac Ganglia for RNA Sequencing and qRT-PCR.*** Left and right PGSNs were dissected from 16-week-old male SHR and age-matched male Wistar rats. Briefly, rats were anaesthetized in an induction chamber (3-5% isoflurane) and humanely killed by a Home Office approved Schedule 1 method: overdose of pentobarbital (Euthatal, 200 mg/mL) and exsanguination. Right stellate ganglia were removed for RNA sequencing and the left stellate ganglia were used for matched *q*RT-PCR experiments. Stellate ganglia were placed in Hanks Buffered Saline Solution without Ca^2+^ and Mg^2+^. For clinical samples, human stellate ganglia were kindly sent by Dr. Ajijola, Dr. Ardell and Dr. Shivkumar from UCLA Cardiac Arrhythmia Center and shipped on dry ice in RNA*later*® RNA Stabilization Solution (ThermoFisher). Rat and human ganglia were cleaned and de-sheathed. Each ganglion was transferred immediately to RLT lysis buffer (Qiagen) with β-mercaptoethanol (1%), and each sample contained tissue from one stellate. Rat stellates were finely chopped and carefully triturated with fire-blown Pasteur pipettes until adequately digested. Human stellates were manually homogenized. RNA was extracted using an RNeasy Mini RNA Extraction Kit (Qiagen) and human RNA was extracted using an RNeasy Maxi RNA Extraction Kit (Qiagen) in accordance with the manufacturer’s instructions. RNA samples were aliquoted for *q*RT PCR and quality control experiments, snap frozen in liquid nitrogen and stored at -80 °C. The RNA quality and integrity from each sample, was confirmed using a 2100 Bioanalyzer Instrument with an RNA picochip (Agilent). Rat samples with an RNA Integrity Number (RIN) less than 8.5 were discarded. Due to difficulties with fast shipping of human samples we accepted human RNA samples with RINs above 5.7. RNA concentrations were determined using a Qubit RNA High Sensitivity Assay Kit (Molecular Probes, Life Technologies) and a Qubit® 2.0 Fluorometer (Invitrogen, Life Technologies.

***cDNA library preparation for RNA Sequencing.*** Four replicate samples containing total RNA extracted from the right sympathetic stellate ganglion of 16-week-old male SHR (n=4) and age-matched Wistar (n=4) were sent to the High-Throughput Genomics Group at the Wellcome Trust Centre for Human Genetics (WTCHG) for RNAseq library construction and sequencing using an Illumina HiSeq 4000 (Illumina, Inc., San Diego, USA). The sequencing libraries were amplified using a SMARTer (first strand synthesis) amplification protocol due to the low initial RNA concentrations obtained from a single stellate and prepared for paired-end sequencing (2 x 75 bp). Each sample was sequenced on two separate lanes to minimize technical error and to increase the sequencing depth (~15-25 million reads per lane). Samples were randomized and blinded to the experimenter. The number of replicates and the sequencing parameters established, were based on recommendations from WTCHG and those published by Conesa et al., 2016^41^.

***Quasi-mapping.*** Transcripts were quantified via the Salmon (version 0.8.2) package using the transcriptome-based quasi-mapping mode^4^. The following commands were used for quasi-mapping in accordance with the Salmon guidelines:
salmon quant –I transcripts_index -l ISR a -1 /SampleX_lane1_mate1.fastq.gz / SampleX_lane2_mate1.fastq.gz -2 / SampleX_lane1_mate2.fastq.gz / SampleX_lane2_mate2.fastq.gz -o Sample1 --dumpEq --posBias --gcBias –writeUnmappedNames. The transcript index used during quasi-mapping was derived from the UCSC refseq rn6.0 mRNA library available at the following link: <http://hgdownload.soe.ucsc.edu/goldenPath/rn6/bigZips/refMrna.fa.gz>.

Data files were assigned alternative names to blind the experimenter during the relevant stages of the quasi-mapping analysis.

***RNAseq Differential Expression Analysis.*** Following sample quantification, the data were imported into R and summarized at the gene-level using the 'Tximport’ function (v1.4.0)^5^. A differential expression analysis of the gene counts for Wistar and SHR samples was performed using the ‘DESeq2’ command in the R package DESeq2 (version 1.16.1)^6^. Significance for differential expression was accepted at the Benjamini-Hochberg adjusted value *p*<0.05. The ‘LFCshrink’ function was used to shrink log_2_ fold change after analysis, for visualization and ranking of genes, as per the DESeq2 vignette^7^.

***cDNA Library Preparation for qRT-PCR.*** 50 ng stellate RNA was obtained for constructing *q*RT-PCR cDNA libraries (n=4/group). For conversion of rat PGSN RNA, the SuperScript^TM^ III VILO^TM^ cDNA synthesis protocol was followed according to manufacturer’s instructions. For conversion of human PGSN RNA, the SuperScript^TM^ IV VILO^TM^ cDNA synthesis protocol was followed according to manufacturer’s instructions (ThermoFisher). The concentration of cDNA in each sample and the 260/280 ratios were calculated (NanoDrop Lite) to detect the presence of contaminants. cDNA samples with an abnormal 260/280 ratio (<1.7 and >1.95) were discarded. The cDNA samples were aliquoted and numerically labelled to blind the experimenter to the rat strain during experimentation. Samples were frozen at -80 °C for long-term storage or retained at 4 °C for immediate use.

***Two-Step Quantitative Real-Time PCR****. qRT*-PCR was used to confirm the presence of the following mRNA transcripts in the PGSN cDNA libraries: β_1_AR (*Adrb1;* Rn00824536_s1, Hs02330048_s1; rat, human respectively), β_2_AR (*Adrb2;* Rn00560650_s1, Hs00240532_s1; rat, human), α_2a_AR (*Adra2a;* Rn00562488_s1, Hs01099503_s1); PAH (*Pah;* Rn00561708_m1, rat), TH (*Th;* Rn00562500_m1, Hs01002182_m1; rat, human), DDC (*Ddc;* Rn01401189_m1, rat), DBH (*Dbh*; Rn00565819_m1, rat), PNMT (*Pnmt;* Rn01495589_g1, Hs01557113_g1; rat, human respectively). The following controls were selected: beta-2-microglobulin (*B2m;* Rn00560865_m1, Hs00187842_m1; rat, human), glyceraldehyde-3-phosphate dehydrogenase (*Gapdh;* Rn99999916_s1, Hs02786624_g1; rat, human). TaqMan® Gene Expression Master Mix (ThermoFisher) was added to each cDNA sample in addition to the selected primer conjugated to a FAM dye and nuclease-free H_2_O. cDNA samples or control samples with no reverse transcriptase were diluted 1:20 for optimal PCR reactions. 20 μL samples (3 repeats) were added to a 96-well plate and run on a real-time quantitative PCR thermocycler (ABI, PRISM). Temperatures were held at 50 °C (2 min) and 95 °C (10 min) before thermal cycling (40 cycles) under the following conditions 95 °C (15 s), 60 °C (1 min). Data displayed in the results section depicts gene counts normalised to *B2m;* however, each primer was analysed against two housekeeping genes, *B2m* and *Gapdh* for validation and accuracy of *q*RT-PCR data. The relative amount of each transcript was calculated using the comparative (C_T_) method (∆∆ C_T_, rat; ∆ C_T_, human)^8^.

***Quantitative Enzyme Linked Immunosorbent Assay (ELISA).*** Protein was extracted from stellate ganglia obtained from 4-week and 20-week male SHR, age-matched Wistar rats and human donors. 100 mg of rat stellate tissue was pooled and homogenized in ice-cold dPBS without Ca^2+^ or Mg^2+^. In alternative experiments, tissue from three human ganglia (80 mg) was separately homogenized in ice-cold dPBS without Ca^2+^ or Mg^2+^. The protein concentration in each sample was quantified using a protein assay (BioRad DC) as per manufacturer’s instructions and the total protein concentration in each sample was normalized. Protein concentrations were established using sandwich ELISAs for β_1_AR (CSB-EL001391RA, CSB-EL001391HU; rat, human respectively), β_2_AR (CSB-EL001392RA, CSB-EL001392HU; rat, human respectively), PNMT (CSB-EL018274RA, CSB-EL018274HU; PNMT rat, human respectively) and TH (CSB-E13102r, CSB-E09661h; rat, human respectively). ELISA assays were performed in accordance with the manufacturer’s instructions (CUSABIO, USA). Background absorbance was measured at 540 nm and subtracted from the values obtained at 450 nm (Infinite F500, TECAN). Absolute concentrations of protein were quantified using a standard curve generated from the supplied standards.

***Live-Cell Förster Resonance Energy Transfer (FRET) Microscopy.*** FRET was employed to monitor real-time changes in intracellular cAMP generation and protein kinase (PKA) activity in randomly selected PGSNs obtained from 4-week preSHR and Wistar rats. Stellate neurons were cultured and transduced with adenovirus particles encoding the respective FRET biosensors Epacs1H187: 5.1 x 10^8^ PFU / mL, AKAR4: 1.3 x 10^9^ PFU / mL) for 24 hours. Experiments were blinded wherever possible, however due to the comprehensive 3-4-day culturing/imaging protocol, it was not possible for a single experimenter to be blinded during the imaging experiments. To detect dynamic changes in cytosolic cAMP or PKA activity, cells were transduced with the loss-of-FRET sensor Epacs1H187^42^ or the gain-of-FRET biosensor A Kinase Activity Reporter 4 (AKAR4)^43^ respectively. FRET biosensor-expressing neurons were imaged 3-4 days post-culture, on an inverted microscope (Nikon) connected to an OptoLED fluorescence imaging system (Cairn Research Ltd., UK). Cells were imaged using 40X or 60X oil-immersion objectives and images were captured with a CoolSNAP HQ2 digital CCD camera (Photometrics). A beam splitter (DV2 Photometrics) included the emission filters for CFP and YFP (ET480/30M, ET535/40M) respectively, and a dichroic mirror (505DCXR). The cells were excited at 430 nm for 100 ms every 15 s. CFP and YFP emission intensities were measured at 480 nm and 535 nm were acquired using Optofluor software (Cairn Research Ltd., UK). For the Epacs1H187 loss-of-FRET sensor, CFP / YFP ratios were calculated. For AKAR4, a gain-of-FRET sensor, YFP / CFP emission intensity ratios were calculated. Background fluorescence was subtracted from emission intensity ratios and data were expressed as intensity per unit time. Mean FRET responses were expressed as the percentage change from baseline (∆*R/R0* where ∆*R = R-R0*. *R0* is the mean ratio over 30 s at baseline in the absence of drug, and R is the mean ratio calculated over 30 s in the presence of drug treatment).

During FRET experiments, cells were perfused continuously with Tyrode’s solution via a gravity-fed perfusion system and the flow rate was controlled at 2-3 mL/min. A stable baseline of at least 2 minutes was required at the start of each experiment. Pharmacological compounds were diluted in Tyrode solution and perfused at the following concentrations: isoprenaline (ISO) 10 nmol/L to 10 μmol/L; β_1_AR agonist dobutamine (DOB) 50 μmol/L; β_1_AR antagonist metoprolol, 100 nmol/L to 10 μmol/L; β_2_AR agonist salbutamol (SAL), 10 μmol/L; β_2_AR agonist ICI 118,551 10 nmol/L to 10 μmol/L. In all experiments, the maximal FRET change of each cell was recorded by exposing the cells to saturating concentrations of an adenylyl cyclase (AC) activator forskolin (FSK) 25 μmol/L and a non-specific PDE inhibitor 3-isobutyl 1-methylxanthine (IBMX) 100 μmol/L, to ensure that the cells responded similarly to the biosensors. In the absence of a FSK/IBMX response, cells were excluded from analysis. The number of cells per group were based on previously-calculated power analyses. For comparisons between cells, the average percentage FRET change was calculated over a 30 s period once equilibrium was established.

***Real-Time Calcium Measurements.*** PGSNs from 4-week preSHR and Wistar rats were dissected, dissociated and incubated for 4-6 hours on the day of culture until they fully adhered to Poly-D-Lysine / laminin-coated coverslips (37°C, 5% CO_2_). To investigate Ca^2+^ responses to ISO, neurons from 4-week Wistar and preSHR were incubated in Indo-1 acetoxymethyl ester (Indo-1AM, ThermoFisher; 2 μmol/L, 45 mins, RT). Stellate neurons loaded with Indo-1AM were imaged on an inverted Nikon Diaphot 200 microscope (Nikon, Tokyo, Japan) and excited at 340 nm with a 100 W Xenon lamp (Nikon, Tokyo, Japan). The emission was split by a dichroic mirror (450 nm) and fluorescence was detected at 405 nm (calcium bound) and 495 nm (calcium free) by two tri-alkali photomultiplier tubes (PMTs, ET Enterprises Ltd., UK) each housed in air cooled enclosures (FACT50, ET Enterprises Ltd., UK) to maintain PMT temperatures at -20 °C. The output from each PMT was integrated in a current-to-voltage converter and digitized at a sampling frequency of 250 Hz (CED 1401). Signals at 405 nm and 495 nm were acquired with Spike2 software and automatically converted into a ratio (495 nm / 405 nm). Recorded values were averaged over 0.5 s intervals providing a final sampling rate of 2 Hz.

During Ca^2+^ imaging experiments a stable baseline was recorded for 30 s prior to stimulation. PGSNs were subsequently stimulated with 50 mmol/L KCl in CO_2_ / HCO_3_ buffered Tyrode solution with an equimolar reduction in NaCl. Upon return to baseline, cells were perfused with isoprenaline (ISO, 1 μmol/L) for 4 minutes and re-stimulated with 50 mmol/L K^+^ in the presence of ISO (1 μmol/L). For control experiments, no ISO was administered. In the absence of a KCl response, cells were excluded from analysis. The values obtained from the Ca^2+^ peak following each KCl stimulation were averaged and baseline values were subtracted from absolute peak size. To determine the effect of the compound on KCl-evoked [Ca^2+^]_i_; the ratio ‘S2 / S1’ was calculated, where S2 was the stimulus in the presence of drug and S1 was the stimulus prior to drug administration. Ratios were compared to time-controlled experiments, where S1 and S2 were both recorded in the absence of drug. Ratiometric data were obtained through conversion of raw data sheets into text files. Fluorescence values were transformed to [Ca^2+^]_i_ concentrations using the following equation derived by Grynkiewicz et al.,^44^:

$$\mathbf{[}{\mathrm{Ca}^{\mathbf{2+}}\mathbf{]}}_{\boldsymbol{i}} \mathbf{=}\boldsymbol{Kd \times\{}\frac{\boldsymbol{sf}}{\boldsymbol{sb}}\boldsymbol{\} \times}\boldsymbol{\{}\frac{\mathbf{(R - R}\mathbf{min}\mathbf{)}}{\mathbf{(R}\mathbf{max}\mathbf{- R)}}\boldsymbol{\}}$$

For control experiments measuring intracellular Ca^2+^, Wistar and WKY neurons were cultured and grown on Poly-D-lysine/laminin-coated coverslips (6 mm, VWR International) and incubated in plating medium (37 °C, 5 % CO_2_) for 36-48 hours. Prior to imaging, cells were incubated in plating media with the ratiometric Ca^2+^ dye Fura-2AM (ThermoFisher; 2 μmol/L, 45 mins, RT). Calcium concentrations were measured at baseline (Tyrode’s, 37 °C) and in response to a KCl challenge (50 mmol/L, equimolar reduction in NaCl, 37 °C). Fura-2AM was excited at 340/380 nm at an interval of 3500 ms. The emitted fluorescence was calculated at 510 nm and converted to Ca^2+^ concentrations using the Grynkiewicz equation as described ^44^.

***High-Pressure Liquid Chromatography Coupled to Electrochemical Detection (HPLC-EC).*** To investigate concentrations of norepinephrine (NE) or epinephrine (Epi) in preSHR compared with Wistar stellates, whole fresh ganglia were dissected from 4-week preSHR (n=4 rats) and 4-week Wistar (n=8 rats). For each experiment, a single ganglion was cleaned, de-sheathed and allowed to recover in bicarbonate-buffered carbogenated Tyrode’s solution (5 min, 37 ^o^C, 95 % O_2_ / 5 % CO_2_). The ganglia were subsequently incubated in bicarbonate-buffered Tyrode’s containing the NET-reuptake inhibitor, desipramine (1 μmol/L, 10 min, 37 ^o^C). Ganglia were electrically stimulated (3 mA, 5 Hz, 5 min); parameters that were adapted from previous electrical stimulation methods^45^. The perfusate was removed and perchloric acid (PCA, 0.1 mol/L) was added at each step to prevent the oxidation of catecholamines. Samples were kept on ice before freezing at -80 ^o^C. At the end of each experiment, whole stellate ganglia were homogenized in bicarbonate-buffered Tyrode’s solution containing PCA (0.1 mol/L), centrifuged (15,000 G, 15 min, 4 ^o^C) and the supernatant stored at -80 ^o^C. For detection and quantification of NE and Epi, the samples were randomized, loaded into an autosampler (JASCO, Model AS-2055/2057) and detected with an isocratic HPLC system. HPLC separation was performed at the flow rate 1 mL / min using a C18 reverse-phase column (CP30710 Microsorb 100-5 C18 S250 x4.6mm, Agilent). The mobile phase (pH 4.7) comprised methanol (11.5% v/v), NaH_2_PO_4_ (120 mmol/L), EDTA (0.8 mmol/L), and sodium octane sulfonate (OSA, 0.5 mmol/L). Concentrations of NE and Epi were quantified using an LC-4B electrochemical detector and a carbon working electrode maintained at +0.7 V versus an Ag/AgCl reference electrode (Decade SDC, Antec). Chromatogram peaks were quantified (area under curve; CLARITY software) and the concentrations of NE were calculated against a 50 mg standard of NE and Epi dissolved in bicarbonate-buffered Tyrode’s containing PCA (0.1 mol/L). In the absence of any NA detection, cells were excluded from analysis.

***Statistical Analysis.*** Data were analyzed using GraphPad Prism software (v6/7). When the data passed normality tests (D'Agostino-Pearson omnibus test or Shapiro-Wilk^46^) unpaired two-tailed Student *t*-tests, or a one or two-way analysis of variance (ANOVA) were used. When the data were not normally distributed, the appropriate nonparametric tests were used with the specific statistical test reported in the figure legend. All data are expressed as the mean ± SEM. Statistical significance was accepted at *p<*0.05 unless otherwise described.

**Supplementary References**

1. Larsen HE, Bardsley EN, Lefkimmiatis K, Paterson DJ. Dysregulation of neuronal Ca^2+^ channel linked to heightened sympathetic phenotype in prohypertensive states. *J Neurosci*. 2016;36(33):8562-8573. doi:10.1523/JNEUROSCI.1059-16.2016.

2. Schindelin J, Arganda-Carreras I, Frise E, et al. Fiji: an open-source platform for biological-image analysis. *Nat Methods*. 2012;9(7):676-682. doi:10.1038/nmeth.2019.

3. Schindelin J, Rueden CT, Hiner MC, Eliceiri KW. The ImageJ ecosystem: An open platform for biomedical image analysis. *Mol Reprod Dev*. 2015;82(7-8):518-529. doi:10.1002/mrd.22489.

4. Patro R, Duggal G, Love MI, Irizarry RA, Kingsford C. Salmon provides fast and bias-aware quantification of transcript expression. *Nat Methods*. 2017;14(4):417-419. doi:10.1038/nmeth.4197.

5. Soneson C, Love MI, Robinson MD. Differential analyses for RNA-seq: transcript-level estimates improve gene-level inferences. *F1000Res*. 2015;4:1521–18. doi:10.12688/f1000research.7563.1.

6. Love MI, Huber W, Anders S. Moderated estimation of fold change and dispersion for RNA-seq data with DESeq2. *Genome Biol*. 2014;15(12):31–21. doi:10.1186/s13059-014-0550-8.

7. Love MI, Huber W, Anders S. Differential analysis of count data – the DESeq2 package. *Genome Biol*. 2014;15(12):31-62. doi:10.1186/s13059-014-0550-8.

8. Schmittgen TD, Livak KJ. Analyzing real-time PCR data by the comparative CT method. *Nat Protoc*. 2008;3(6):1101-1108. doi:10.1038/nprot.2008.73.

**Supplementary Results**

| **Table S1** | | |
| --- | --- | --- |
| **Characteristics of Human Donors** | | |
| **Donor** | **Gender** | **Complications** |
| **#19** | Male | Non-ischemic cardiomyopathy, ventricular fibrillation, LVEF 30-35% |
| **#23** | Male | None noted |
| **#24** | Male | None noted |

| **Figure S1** | | | | | | | |
| --- | --- | --- | --- | --- | --- | --- | --- |
| 1. **Evoked-Ca^2+^: Rat** | | | 1. **ICC Negative Controls** | | | | |
| **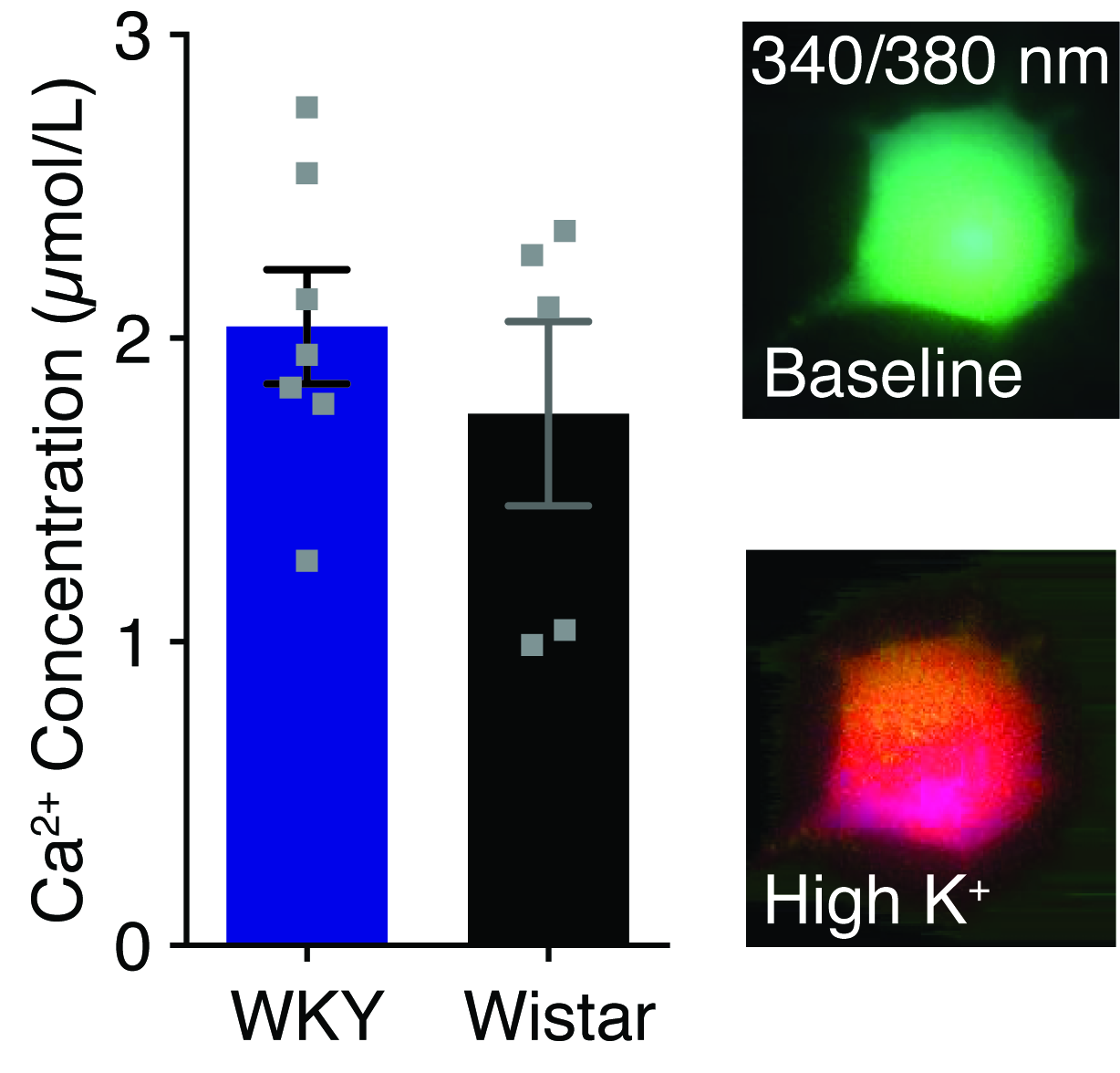** | | | **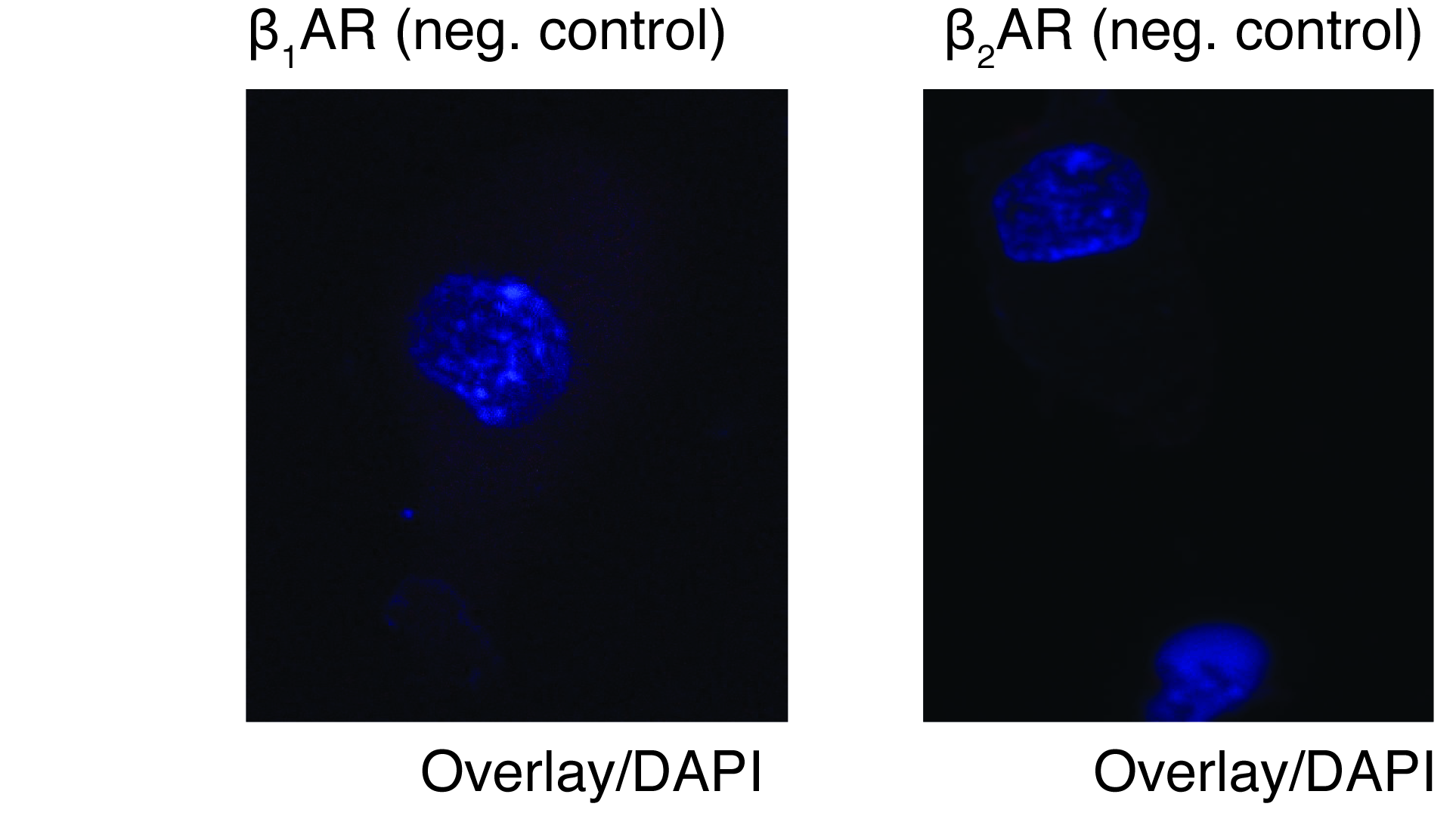** | | | | |
| 1. **RNAseq Fold Change: Adult Rat** | 1. ***q*RT-PCR:**   **Young and Adult Rat** | | | | 1. ***q*RT-PCR:**   **Human** | | |
| **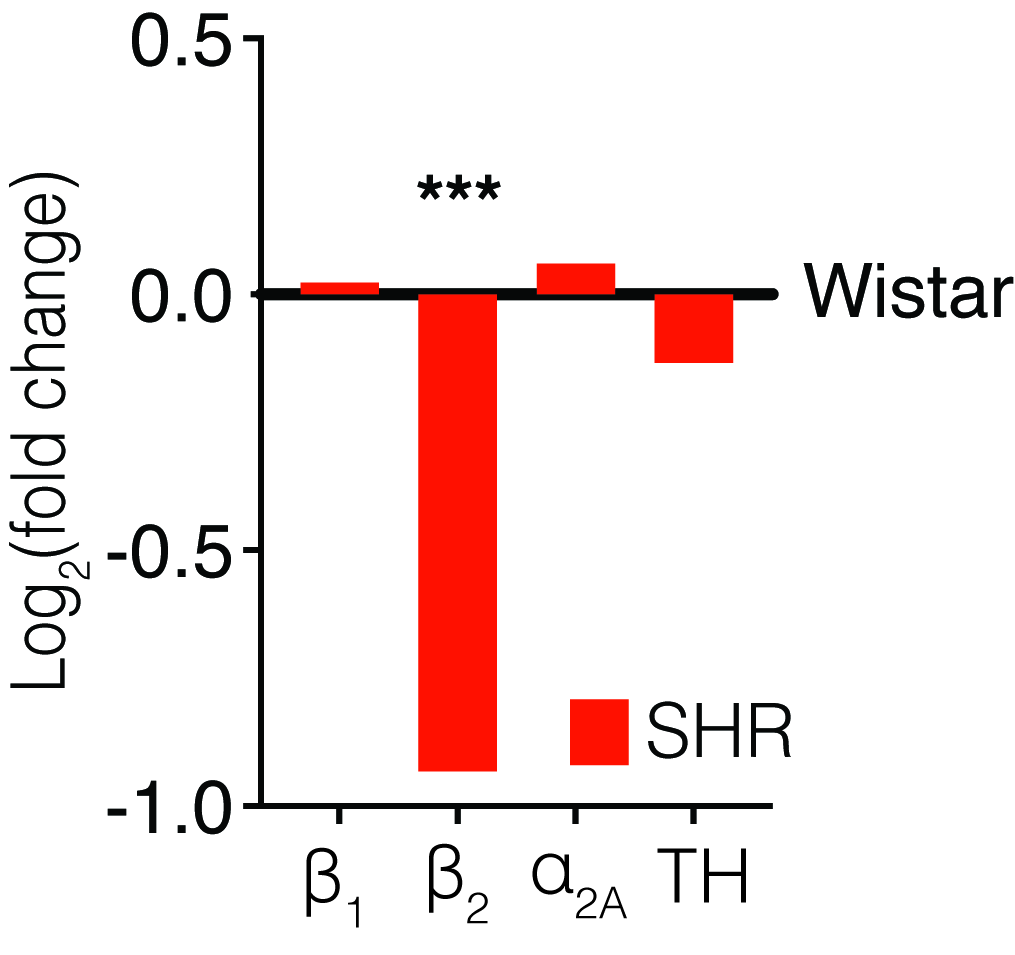** | **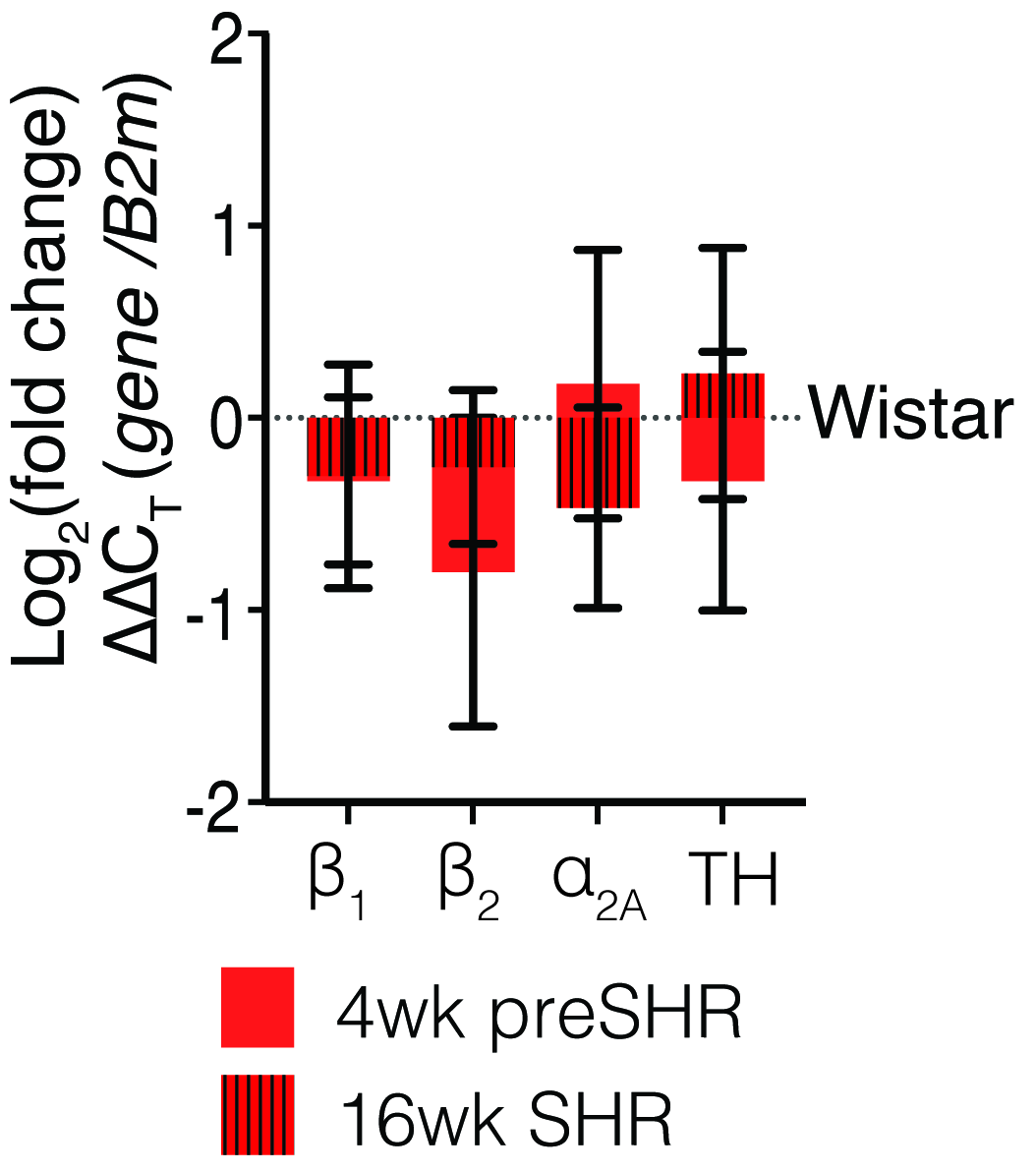** | | | | **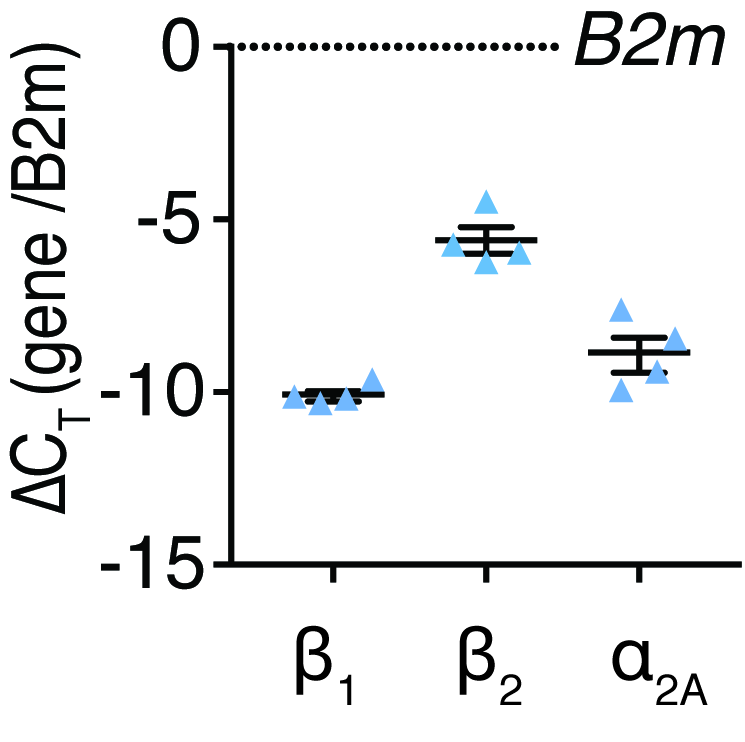** | | |
| **S1.** There was no difference in sympathetic Ca^2+^ responses to 50 mmol/L between PGSNs obtained from 16-week Wistar or WKY rats. Given the absence of a Ca^2+^ phenotype, Wistar rats were used as the control in this study (A). For immunocytochemistry (ICC) experiments, the absence of staining with the primary antibody omitted was used as a control for nonspecific binding of the secondary antibody (B). The transcriptome of the sympathetic stellate ganglia was sequenced in 16-week old male Wistar rats (n=4) and age-matched SHR (n=4). Using RNAseq (C) and *q*RT-PCR (D) we identified the presence of β_1_AR (*Ardb1*) and β_2_AR (*Ardb2*) mRNA transcripts in addition to tyrosine hydroxylase (*Th*) and α_2A_AR (*Adra2a*) mRNA transcripts. In the RNAseq dataset (C), *Ardb2* expression was significantly lower in SHR ganglia compared with Wistar (p.adj = 0.00945; Salmon-DESeq2 method^6^). Data points represent log_2_ (fold change) ± SEM. *q*RT-PCR validated the presence of transcripts in RNA extracted from 4-week (n=3/group) and 16-week rats (n=4/group). Fold changes were calculated by the ∆∆C_T_ method^8^, where the difference in counts between the gene of interest and the housekeeping gene (*B2m*) was calculated (∆). The difference between the two strains (∆∆) was quantified and depicted as log_2_ (fold change) ± SEM. There was no significant difference in the levels of mRNA for *Adrb1, Adrb2,* *Adra2a* or *Th* between strains or between age groups (D). In stellate ganglia obtained from human donors (E), *q*RT-PCR confirmed mRNA transcripts encoding β_1_AR (Adrb1) and β_2_AR (*Adrb2*). Human stellate ganglia were also α_2A_AR positive (*Adra2a*). *q*RT-PCR data were analyzed by the ∆C_T_ method^8^ where data represents the difference (∆) in counts relative to the housekeeping gene *B2m* (E). Individual data points represent an average (of 3 technical replicates) from one human stellate (3 patients, 4 stellates, un-pooled samples). | | | | | | | |
| **Figure S2** | | | | | | | |
| 1. **EpacH187**   **Raw Fluorescence Example Trace: Rat** | | | 1. **AKAR4**   **Raw Fluorescence Example Trace: Rat** | | | | |
| 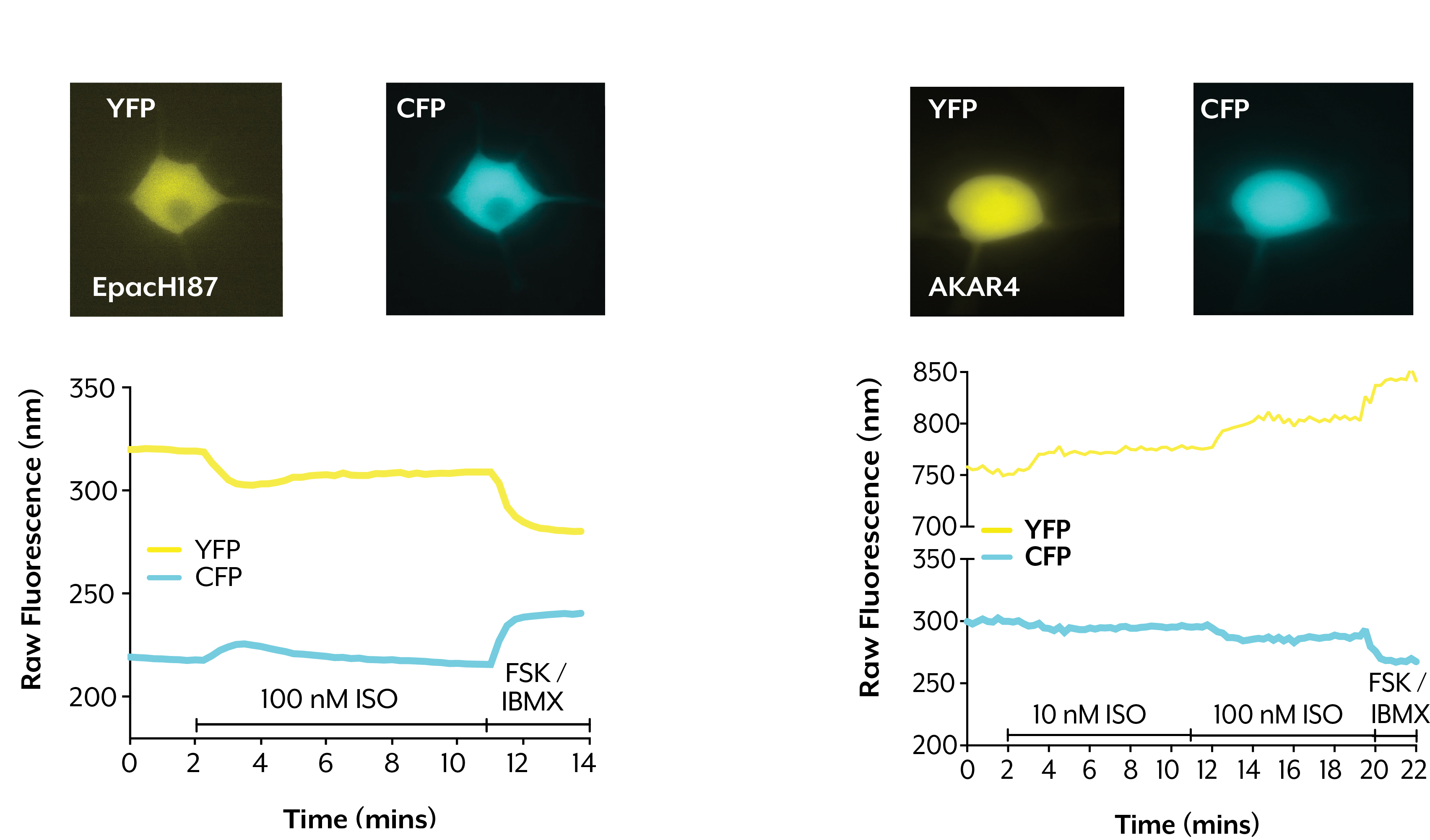 | | | 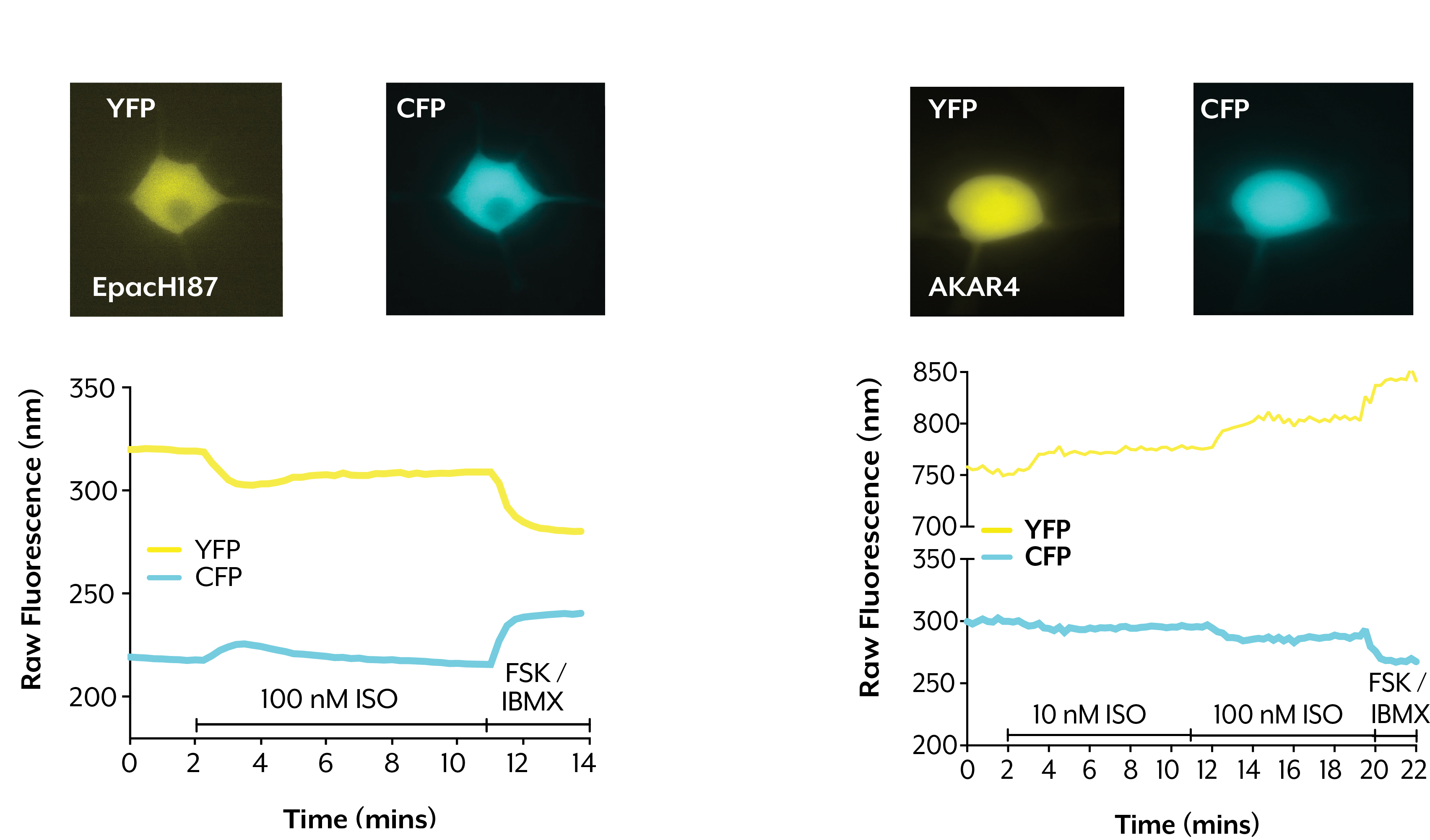 | | | | |
| 1. **Ca^2+^ Imaging Time Controlled Experiment Example Trace: Rat** | | | | | | | |
|    | | | | | | | |
| **S2.** Example YFP and CFP fluorescence traces over-time, as emitted from the cytosolic FRET sensors: EpacH187 (A) and AKAR4 (B) in response to ISO (10-100 nmol/L). Live Ca^2+^ imaging was conducted on PGSNs obtained from 4-week control and preSHR PGSNs with Indo-1AM. A time-controlled raw fluorescence Ca^2+^ trace is displayed (C). In time-controlled experiments, Wistar and preSHR PGSNs (n= 5, 6 respectively) were exposed to two KCl stimulations (50 mmol/L; S1, t = 0.25 mins; S2, t = 4 mins). There was no significant difference in Ca^2+^ responses to high K^+^ between strains, or between stimulation 1 and 2 independent of strain. | | | | | | | |
| **Figure S3** | | | | | | |  |
| 1. **RNAseq Fold Change: Adult Rat** | | | | | 1. ***q*RT-PCR: Adult Rat** | |  |
| **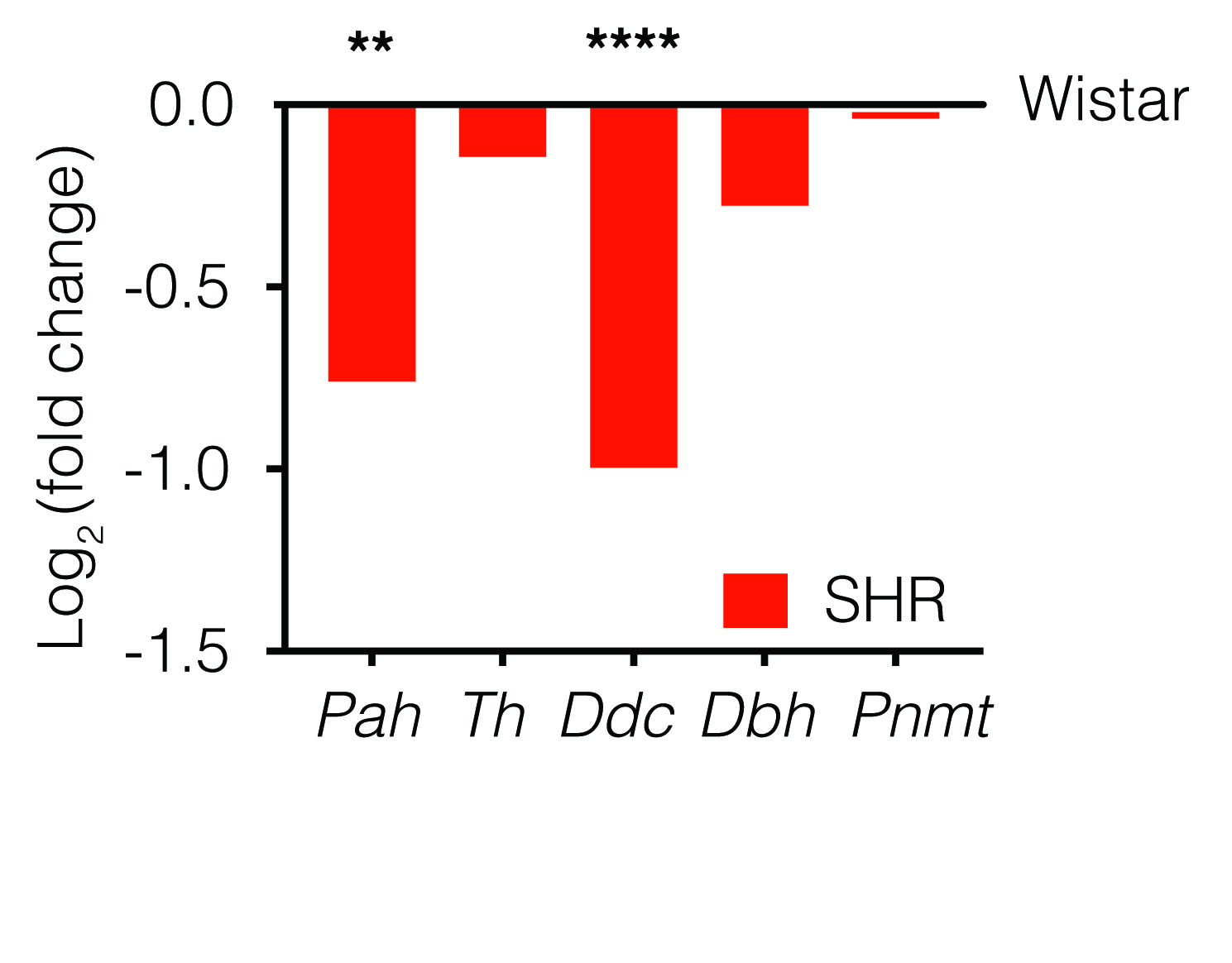** | | | | | ***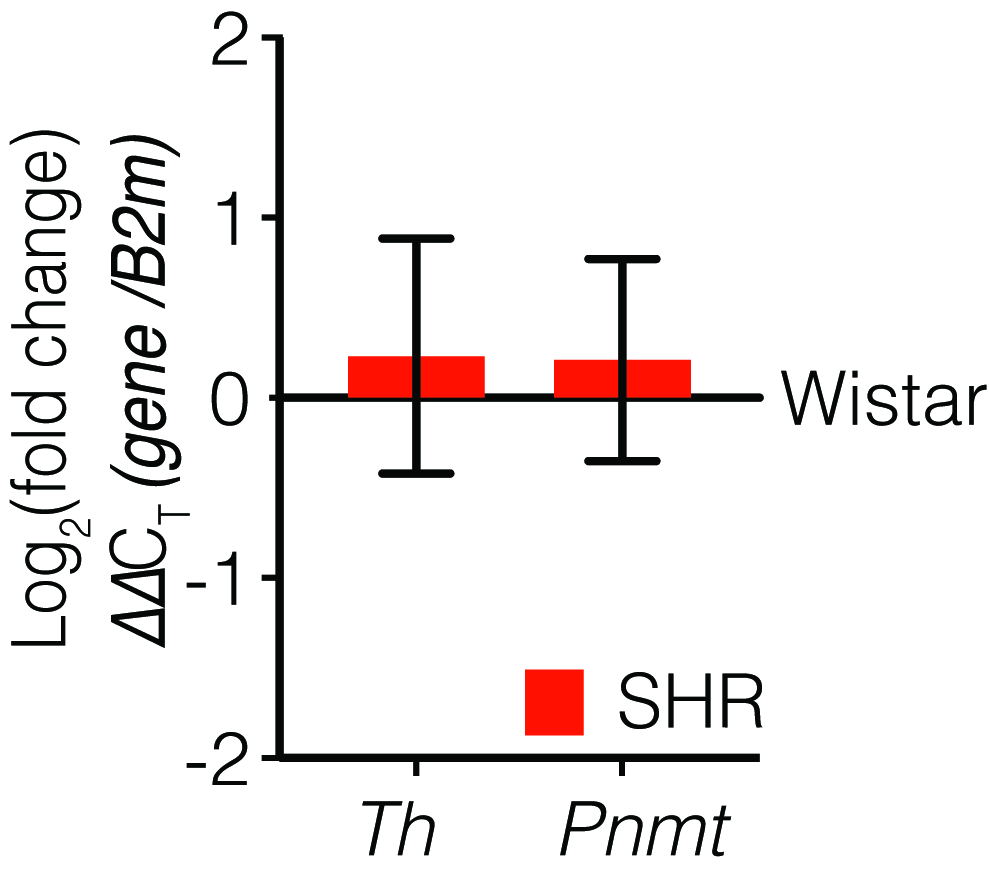*** | |  |
| 1. ***q*RT-PCR: Human** | | | | | | |  |
| ***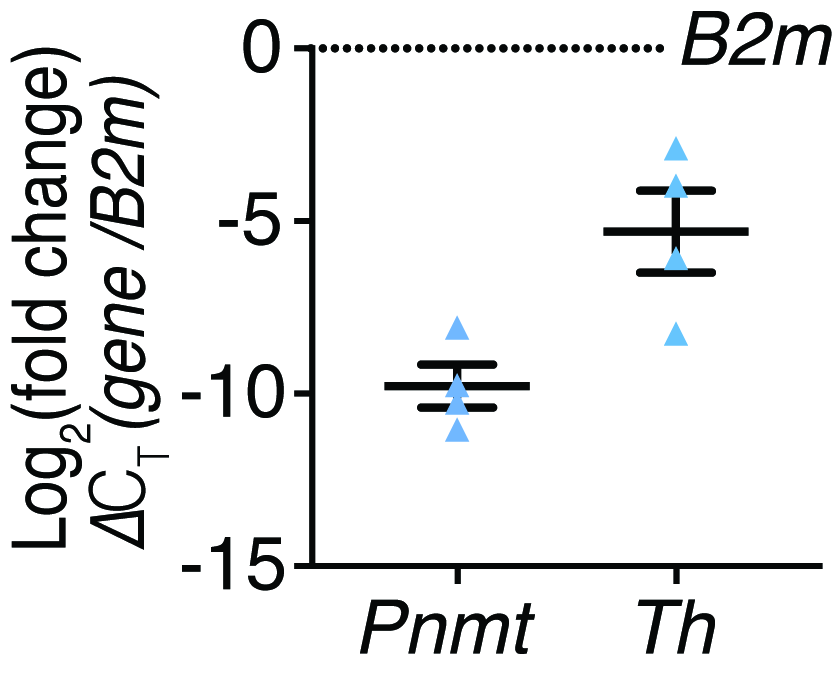*** | | | | | | |  |
| **S3.** Using RNAseq we identified the mRNA transcripts encoding enzymes required for norepinephrine (NE) synthesis: phenylalanine hydroxylase (*Pah*), Tyrosine Hydroxylase (*Th*), L-DOPA decarboxylase (*Ddc*), Dopamine β-hydroxylase (*Dbh)* and Phenylethanolamine-N-methyltransferase (*Pnmt*); the enzyme required for the conversion of NE to epinephrine (Epi). *Pah* and *Ddc* transcripts are significantly lower in SHR PGSNs (*p.*adj=0.0719 (*Pah*); 6.64 x 10^-15^(*Ddc*); Salmon-DESeq2 method^6^). mRNA transcripts are represented as Log_2_(Fold change) ± SEM (A). The presence of *Pnmt* and *Th* was confirmed by *q*RT-PCR in PGSNs from 4-week and 16-week Wistar and SHR. Data were normalized to *B2m* using the ∆∆C_T_ method^8^ and expressed as Log_2_ (Fold change) ± SEM (B). In human stellates, *q*RT-PCR confirmed the presence of *Th* and *Pnmt* (C). Data were normalized to a control housekeeping gene *B2m* using the ∆C_T_ method^8^ where the data represents the difference (∆) in counts relative to the house keeping gene ± SEM (C). Individual data points represent an average (of 3 technical replicates) from one human stellate (3 patients, 4 stellates, un-pooled samples). | | | | | | |  |
